# Effect of Digital Medication Event Reminder and Monitor-Observed Therapy vs Standard Directly Observed Therapy on Health-Related Quality of Life and Catastrophic Costs in Patients With Tuberculosis: A Secondary Analysis of a Randomized Clinical Trial

**DOI:** 10.1001/jamanetworkopen.2022.30509

**Published:** 2022-09-15

**Authors:** Tsegahun Manyazewal, Yimtubezinash Woldeamanuel, Abebaw Fekadu, David P. Holland, Vincent C. Marconi

**Affiliations:** 1 Center for Innovative Drug Development and Therapeutic Trials for Africa, College of Health Sciences, Addis Ababa University, Addis Ababa, Ethiopia; 2 Emory University School of Medicine and Rollins School of Public Health, Atlanta, Georgia

## Abstract

**Importance:**

Little is known about whether digital adherence technologies are economical for patients with tuberculosis (TB) in resource-constrained settings.

**Objective:**

To test the hypothesis that for patients with TB, a digital medication event reminder monitor (MERM)-observed therapy provides higher health-related quality of life (HRQoL) and lower catastrophic costs compared with standard directly observed therapy (DOT).

**Design, Setting, and Participants:**

This study was a secondary analysis of a randomized, 2-arm, open-label trial conducted in 10 health care facilities in Ethiopia. Eligible participants were adults with new or previously treated, bacteriologically confirmed, drug-sensitive pulmonary TB who were eligible to start first-line anti-TB therapy. Participants were enrolled between June 2, 2020, and June 15, 2021, with the last participant completing follow-up on August 15, 2021.

**Interventions:**

Participants were randomly assigned (1:1) to receive a 15-day TB medication supply dispensed with a MERM device to self-administer and return every 15 days (intervention arm) or the standard in-person DOT (control arm). Both groups were observed throughout the standard 2-month intensive treatment phase.

**Main Outcomes and Measures:**

Prespecified secondary end points of the original trial were HRQoL using the EuroQoL 5-dimension 5-level (EQ-5D-5L) tool and catastrophic costs, direct (out-of-pocket) and indirect (guardian and coping) costs from the individual patient perspective using the World Health Organization’s Tool to Estimate Patient Costs, and common factors associated with lower HRQoL and higher catastrophic costs.

**Results:**

Among 337 patients screened for eligibility, 114 were randomly assigned, and 109 were included in the final complete-case intention-to-treat analysis (57 control and 52 intervention participants). The mean (SD) age was 33.1 (11.1) years; 72 participants (66.1%) were men, and 15 (13.9%) had HIV coinfection. EQ-5D-5L overall median (IQR) index value was 0.964 (0.907-1). The median (IQR) value was significantly higher in intervention (1 [0.974-1]) vs control (.908 [0.891-0.964]) (*P* < .001). EQ-5D-5L minimum and maximum health state utility values in intervention were 0.906 and 1 vs 0.832 and 1 in control. Patients’ overall median (IQR) postdiagnosis cost was Ethiopian birr (ETB) 80 (ETB 16-ETB 480) (US $1.53). The median cost was significantly lower in intervention (ETB 24 [ETB 16-ETB 48]) vs control (ETB 432 [ETB 210-ETB 1980]) (*P* < .001), with median possible cost savings of ETB 336 (ETB 156-ETB 1339) (US $6.44) vs the control arm. Overall, 42 participants (38.5%; 95% CI, 29.4%-48.3%) faced catastrophic costs, and this was significantly lower in the intervention group (11 participants [21.2%]; 95% CI, 11.1%-34.7%) vs control (31 participants [54.4%]; 95% CI, 40.7%-67.6%) (*P* < .001). Trial arm was the single most important factor in low HRQoL (adjusted risk ratio [ARR], 1.49; 95% CI, 1.35-1.65; *P* < .001), while trial arm (ARR, 2.55; 95% CI, 1.58-4.13; *P* < .001), occupation (ARR, 2.58; 95% CI, 1.68-3.97; *P* < .001), number of cohabitants (ARR, 0.64; 95% CI, 0.43-0.95; *P* = .03), and smoking (ARR, 2.71; 95% CI, 1.01-7.28; *P* = .048) were the most important factors in catastrophic cost.

**Conclusions and Relevance:**

In patients with TB, MERM-observed therapy was associated with higher HRQoL and lower catastrophic costs compared with standard DOT. Patient-centered digital health technologies could have the potential overcoming structural barriers to anti-TB therapy.

**Trial Registration:**

ClinicalTrials.gov Identifier: NCT04216420

## Key Points

**Question:**

Does a digital medication event reminder monitor–observed therapy for patients with tuberculosis result in higher health-related quality of life compared with the standard directly observed therapy?

**Findings:**

In this secondary analysis of a randomized clinical trial involving 109 adults with pulmonary tuberculosis, health-related quality of life was significantly better in patients who used the digital medication event reminder monitor system compared with those receiving standard directly observed therapy. Patients using the digital therapy also had significantly lower rates of catastrophic costs.

**Meaning:**

These results suggest that digital health technologies may improve quality of life and reduce catastrophic costs among patients with pulmonary tuberculosis, in particular those who face structural barriers to standard therapy.

## Teaser

This randomized clinical trial compares health-related quality of life scores for patients with tuberculosis who had therapy monitored and tracked with an electronic device vs daily in-person visits for medication.

## Introduction

Tuberculosis (TB) remains a disease of poverty that disproportionately affects the world's most economically vulnerable populations. Although highly efficacious, current anti-TB treatments are very costly for patients and their households as they have to cover costs associated with facility-based medication pick up, imposing significant challenges to treatment adherence and resulting in treatment failure, drug resistance, and continuous disease transmission.^1,2^ Current anti-TB treatments last at least 6 months for drug-susceptible TB and 20 months for drug-resistant TB, with patients swallowing medications at a health care facility under directly observed therapy (DOT) throughout the intensive phase in most high-burden countries. This leads patients and their households to be overburdened with structural barriers to therapy adherence, including catastrophic costs and income losses resulting from transportation, food, and accommodation for in-person DOT that they are incapable of avoiding, which contradicts the World Health Organization (WHO)’s End TB strategy to shift the percentage of TB-affected families facing catastrophic costs to 0%. Efforts are currently under way to develop short-course anti-TB regimens^3,4^ and cost-effective digital adherence technologies (DATs) as alternatives to DOT.^5,6^

An effectiveness-implementation type 2 hybrid, randomized controlled trial was designed to assess whether a digital medication event reminder monitor (MERM)-observed self-administered therapy is effective for patients with TB compared with the standard in-person DOT in a high-burden, resource-constrained context like in Ethiopia. In the protocol, the co-primary end points were the level of treatment adherence (individual-level percentage adherence over the 2-month intensive phase measured by adherence records compiled from a MERM device vs DOT records) and sputum conversion (participant with sputum smear converted following the standard 2-month intensive phase treatment). The trial protocol^7^ and a systematic review in support of the trial^8^ have been published previously. This manuscript describes prespecified secondary outcomes of the trial: health-related quality of life (HRQoL), catastrophic costs, and postdiagnostic costs from an individual patient’s perspective. The MERM device (Wisepill Technologies) holds an electronic module and a medication container to record adherence to treatment, store medication, emit audible alerts and color-coded visual light cues (ie, green, yellow, and red) to remind patients to swallow and refill their medication, and enable clinicians to monitor adherence digitally.^9^ The device is described further in the trial protocol. Some studies have demonstrated improved TB care with the use of electronic medication event monitors,^10,11,12,13^ but results were inconsistent and only a few followed a randomized trial design.

Generating evidence on the effects of such digital health technologies on HRQoL and patient cost of treatment within vastly different country contexts is equally important to ensure patients leverage the benefits. The WHO Digital Health Strategy 2020-2025 considered cost-effectiveness as one of the major arguments for the appropriate use of digital health.^14^ Studies carried out in sub-Saharan African countries, including Eritrea,^15^ Nigeria,^16^ South Africa,^17^ Zimbabwe,^18^ and Ethiopia^19^ reported a strong inverse association between TB and patient HRQoL. However, studies that have described HRQoL and the economic benefits of the MERM device for patients with TB are scarce, and to the best of our knowledge, no study has been carried out in sub-Saharan Africa and no randomized controlled studies have been documented. Thus, this study aimed to test the hypothesis of a prespecified secondary outcome that MERM-observed self-administered therapy improves the HRQoL and reduces catastrophic costs for patients with TB compared with the standard DOT.

## Methods

### Study Design

This study describes prespecified secondary outcomes of a multicenter, 2-arm, randomized, attention-controlled, effectiveness-implementation type 2 hybrid trial in 10 health care facilities in Ethiopia. The trial is registered with ClinicalTrials.gov (NCT04216420), and a full description of the study was provided elsewhere (Supplement 1).^7^ Reporting of the trial follows the Consolidated Standards of Reporting Trials (CONSORT) reporting guideline.

The protocol was approved by the Ethiopian Food and Drug Authority, the Ethiopian National Research Ethics Review Committee, the institutional review board of the College of Health Sciences at Addis Ababa University, and the Ethical Clearance Committee of the Ababa Health Bureau. All participants provided written informed consent. A standard operating procedure was developed for each activity and placed at each study site following the review and approval by the implementing clinicians.

### Participants

Eligible potential patients were adults aged 18 years or older; had new or previously treated, bacteriologically confirmed drug-sensitive pulmonary TB; were eligible to start the standard WHO-approved fixed-dose combination of 6-month first-line anti-TB medication; and from the outpatient setting. Patients were ineligible if they had known drug-resistant TB or if they had a concurrent health condition that precluded informed consent or safely participating in the study procedures.

### Randomization and Masking

Consented participants were randomly assigned (1:1) to either MERM-enabled self-administered therapy (intervention arm) or DOT standard care (control arm) using a computer-generated random number sequence developed by a trial expert who did not participate in patient recruitment. Permuted block randomization method was used to randomly allocate participants and maintain a balance of the number of participants. The study investigators who were responsible for assessing study outcomes and writing the report were masked to group allocation until the manuscript was completed. Because of the scope of the trial, participants and the other study staff were not masked to group allocation. Stratification was not needed for key variables. A statistician masked to group allocation performed the analyses.

### Intervention

Treatment follow-up was made by the full-time clinicians in the TB clinic following a moderate on-site orientation, and the MERM software was set up on computers that had already been in use in TB clinics or similar facilities to understand the sustainability of the intervention in a broad sense. For both arms, baseline information, including demographic, socioeconomic, behavioral, and social characteristics were collected using a semi-structured questionnaire. Both arms were followed throughout the intensive treatment phase lasting 2 months for drug-susceptible TB. Treatment was based on the WHO-recommended 2-month fixed-dose combination of first-line anti-TB drugs delivered as a single daily dose, ie, 2RHZE (150 mg of rifampicin [R], 75 mg of isoniazid [H], 400 mg of pyrazinamide [Z], and 275 mg of ethambutol [E]).

Participants in the intervention arm were informed on how to use the MERM device and given an instructional leaflet with patient-friendly explanatory graphics prepared in the national language that outlined the procedures. Thereafter, participants received a 15-day TB medication supply (HRZE fixed-dose combination therapy of 15 doses) in the MERM device to self-administer. Participants returned to the clinic every 15 days, at which point a clinician counted any remaining tablets in the pillbox and connected the MERM module with the computer. Along with the participant, the clinician downloaded pill-taking data from the device to the computer and reviewed the event reports over the previous 15 days. Any missed event where no ingestion occurred over a particular prescribed ingestion period in the event report was evaluated against any remaining tablets in the pillbox and discussed further with the participant for confirmation.

Participants in the control arm were managed according to the standard DOT practice, where they visited the health care facility each day throughout the 2-month intensive phase to swallow their daily dose of RHZE with direct observation by TB clinicians. The continuation phase (4 months) followed the standard DOT practice for both arms.

### Estimates of HRQoL

HRQoL was measured at the end of the intensive phase using the EuroQoL 5-Dimension 5-Level Questionnaire (EQ-5D-5L),^20^ which was administered by trained study staff. The tool measures health across 5 domains: mobility, self-care, ability to do usual activities, pain or discomfort, and anxiety or depression. Each domain has 5 levels of response—no problems, slight problems, moderate problems, severe problems, and extreme problems or inability—that provide a descriptive profile used to generate a health state utility value. Health state index scores generally range from less than 0 (where zero is the value of a health state equivalent to death; negative values representing values worse than death based on patient perception) to 1 (the value of full health), with higher scores indicating higher health utility states.^21^ Patient responses were converted into a health state, a 5-digit number generated by concatenating each of the 5 responses to provide a single index value score that determines the health state (eg, full health would be coded 11111). Health state index scores were calculated from individual health profiles using the Ethiopia value set.^22^

### Estimates of Costs

Individual patient cost of treatment was measured at the end of the intensive phase using the Tool to Estimate Patients’ Costs developed by the WHO, KNCV Tuberculosis Foundation, and the Japan Anti-Tuberculosis Association,^23^ which was adapted for the trial and administered by trained study staff. The tool is designed to assess how TB affects the welfare of households and individuals by estimating the costs that patients with TB and their households incur before and during diagnosis and during treatment of TB. The tool subdivides the direct and indirect medical costs incurred into 3 stages: prediagnostic (before diagnosis), diagnostic (during diagnosis or pretreatment), and treatment (during treatment) costs. In general, there were no fixed or marginal costs required from patients. Because the prediagnostic and diagnostic costs were similar for the 2 arms, data were collected on costs during treatment with a focus on costs related to anti-TB drug pick-up, guardian costs, and coping costs over the 2-month intensive phase. The anti-TB drug pick-up cost determined costs for travel, food and accommodation, frequency of travel, length of stay, and distance from the facility. The guardian cost captured data to determine if participants were going with guardians and if so, the reason and frequency. The coping costs were costs to meet daily living requirements despite the extra expenditures or loss of income, which could include selling assets, borrowing money, availing loans, incurring another job to cover the costs, or removing children from school. Costs for anti-TB medications were not included as these were fully covered by the Ethiopian government for all patients with TB.

### Primary and Secondary Outcomes

The main objective of this prespecified secondary analysis was to evaluate the association between MERM-observed therapy and HRQoL, where the HRQoL was measured and calculated for each participant by arm using the EQ-5D-5L score ranging from 0 to 1, with a higher score designating better HRQoL. The secondary outcomes were: (1) catastrophic cost (patients with overall TB treatment cost exceeding or equivalent to 20% of their income over the 2-month treatment period); (2) postdiagnostic cost from an individual patient’s perspective, including cumulative direct costs (out-of-pocket costs related to anti-TB drug pick-up) and indirect costs (guardian and coping costs) over the 2-month intensive phase, and (3) factors contributing to lower HRQoL (having at least 1 health problem) and higher catastrophic costs.

### Statistical Analysis

Analysis was based on the complete-case intention-to-treat (ITT) principle. Descriptive statistics including frequency and percentage were used to describe the health state of the study participants. Multiple bar charts with cross-tabulation were used to illustrate distributions of health states by study arms. χ^2^ and Fisher exact tests were employed to compare the 5 EQ-5D-5L health domains by study arms. Both Kolmogorov-Smirnov and Shapiro–Wilk tests revealed that the distribution of EQ-5D-5L index value (ie, the health state utility) was not normal. Median values were used to summarize EQ-5D-5L index value. A nonparametric Mann-Whitney U test was employed to compare the difference in EQ-5D-5L index value among study arms. A log-binomial model was used to identify risk factors for lower HRQoL, which was having at least 1 health problem.

The overall TB treatment cost was estimated by considering costs related to anti-TB drug pick-up, guardian costs, and coping costs over the 2-month intensive phase. Both Kolmogorov-Smirnov and Shapiro-Wilk tests revealed that the distribution of overall TB treatment cost was non-normal, therefore, median values with IQR were used to summarize costs. A nonparametric Mann-Whitney U test was employed to compare cost differences among study arms. The proportion of study participants who faced catastrophic costs at a cut-off point of 20% was estimated. A cross-tabulation was employed to evaluate the distribution of catastrophic cost over the study arms and a χ^2^ test was used to test the association between catastrophic cost and study arms.

The sample size was calculated considering a 2-sided type I error of 5%, a power of 80%, 20% attrition rate, 20% noninferiority margin, a delta of 0.63, and a continuous outcome of percentage adherence over the 2-month intensive phase, with a standard deviation of 41% and 79% of average adherence. The results yielded a sample size of 57 in each arm for a total of 114 participants.

## Results

### Participant Characteristics

Recruitment took place between June 2, 2020, and June 15, 2021, with the last participant completing follow-up on August 15, 2021. Three hundred thirty-seven patients were screened for eligibility and 114 of these were included and randomly assigned into the trial 1:1 allocation ratio. In the final complete-case ITT analysis, 109 consented participants were included, 52 from the intervention arm and 57 from the control arms (Figure). The imbalance resulted from 4 participants being transferred and 1 lost to follow-up from the intervention arm. Imputations were not considered as the percentage of missing data was below 5%.

**Figure.**
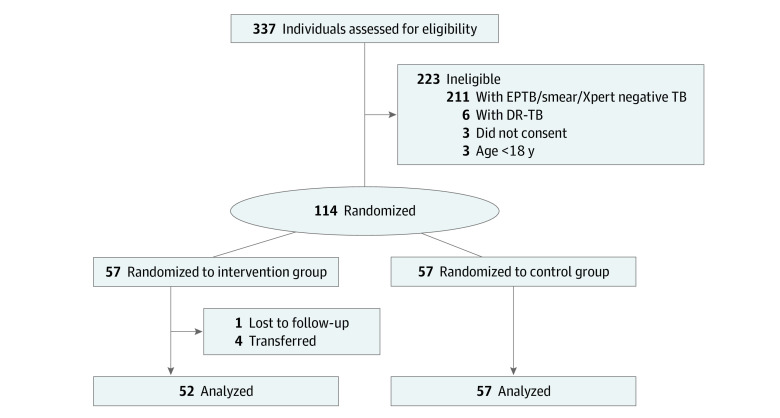
Flowchart of Trial Participation Abbreviations: DR-TB, drug-resistant tuberculosis; EPTB, extrapulmonary tuberculosis.

Of the 109 participants analyzed, the mean (SD) age was 33.1 (11.1) years; 72 participants (66.1%) were men, 6 (5.5%) were currently homeless, 68 (62.4%) lived in a house with a single bedroom, and 18 (17.4%) smoked cigarettes (Table 1). Eleven (10.1%) were re-treatment cases and had completed their previous treatment, and 15 (13.9%) had HIV coinfection, of whom 11 (73.3%) were receiving antiretrovirals. Laboratory diagnostic tools for pretreatment confirmation of TB were a MTB/RIF (mycobacterium tuberculosis/resistance to rifampicin) assay (68 participants [62.4%]) and acid-fast bacillus smear microscopy (41 participants [37.6%]). Mean (SD) monthly income was US $67.50 ($71.20; range, $0-$333.30). Baseline characteristics and HIV status were balanced between the 2 groups.

**Table 1.** Characteristics of Study Participants

Variables	Participants, No. (%)		
	Total (N = 109)	Intervention (n = 52)	Control (n = 57)
Sex			
Women	37 (33.9)	15 (28.8)	22 (38.6)
Men	72 (66.1)	37 (71.2)	35 (61.4)
Marital status			
Never	50 (45.9)	23 (44.2)	27 (47.4)
Married	50 (45.9)	28 (53.8)	22 (38.6)
Widowed	2 (1.8)	0	2 (3.5)
Divorced	7 (6.4)	1 (1.9)	6 (10.5)
Occupation status			
No job	22 (20.2)	9 (17.3)	13 (22.8)
Student	4 (3.7)	4 (7.7)	0
Farmer	1 (0.9)	0	1 (1.8)
Trader	10 (9.2)	5 (9.6)	5 (8.8)
Housewife	9 (8.3)	2 (3.8)	7 (12.3)
Government employee	10 (9.2)	5 (9.6)	5 (8.8)
Daily laborer	42 (38.5)	19 (36.5)	23 (40.4)
Other	11 (10.1)	8 (15.4)	3 (5.3)
Highest level of education			
No formal education	9 (8.3)	3 (5.8)	6 (10.5)
Primary	41 (37.6)	22 (42.3)	19 (33.3)
Secondary	28 (25.7)	12 (23.1)	16 (28.1)
Preparatory	11 (10.1)	5 (9.6)	6 (10.5)
University diploma	9 (8.3)	4 (7.7)	5 (8.8)
University diploma or above	11 (10.1)	6 (11.5)	5 (8.8)
Residential status			
Lives alone	13 (11.9)	5 (9.6)	8 (14.0)
Lives with family	83 (76.1)	42 (80.8)	41 (71.9)
Lives with friends	5 (4.6)	1 (1.9)	4 (7.0)
Homeless	6 (5.5)	3 (5.8)	3 (5.3)
Other	2 (1.8)	1 (1.9)	1 (1.8)
No. of cohabitants			
≤3	63 (57.8)	32 (61.5)	31 (54.4)
4-6	37 (33.9)	16 (30.8)	21 (36.8)
7-9	7 (6.4)	2 (3.8)	5 (8.8)
≥10	2 (1.8)	2 (3.8)	0
Bedrooms			
1	68 (62.4)	33 (63.5)	35 (61.4)
2	30 (27.5)	14 (26.9)	16 (28.1)
3	6 (5.5)	3 (5.8)	3 (5.3)
≥4	5 (4.6)	2 (3.8)	3 (5.3)
Household head			
Yes	61 (56.0)	31 (59.6)	30 (52.6)
No	48 (44.0)	21 (40.4)	27 (47.4)
Residency status			
Permanent	89 (81.7)	40 (76.9)	49 (85.9)
Temporary	20 (18.3)	12 (23.1)	8 (14.0)
Smoking, No./d			
Never	90 (82.6)	46 (88.5)	44 (77.2)
1-5	18 (16.5)	5 (9.6)	13 (22.8)
6-10	1 (0.9)	1 (1.9)	0
Khat (a stimulant)			
Never	87 (79.8)	43 (82.7)	44 (77.2)
1/wk	10 (9.2)	3 (5.8)	7 (12.3)
≥2/wk	8 (7.3)	4 (7.7)	4 (7.0)
1/mo	4 (3.7)	2 (3.8)	2 (3.6)
Alcohol			
Never	67 (61.5)	35 (67.3)	32 (56.1)
>1/d	12 (11.0)	5 (9.6)	7 (12.3)
2-5/d	19 (17.4)	7 (13.5)	12 (21.1)
≥6/d	3 (2.8)	1 (1.9)	2 (3.5)
Rarely	8 (7.3)	4 (7.7)	4 (7.0)
HIV^a^			
Negative	93 (86.1)	44 (84.6)	49 (87.5)
Positive	15 (13.9)	8 (15.4)	7 (12.5)
On antiretroviral (if HIV positive)			
Yes	11 (73.3)	6 (75.0)	5 (71.4)
No	4 (26.7)	2 (25.0)	2 (28.6)
TB treatment			
New	98 (89.9)	46 (88.5)	52 (91.2)
Relapse	11 (10.1)	6 (11.5)	5 (8.8)
Place of diagnosis			
Study facility	75 (68.8)	33 (63.5)	42 (73.7)
Health center	3 (2.8)	1 (1.9)	2 (3.5)
Public hospital	10 (9.2)	8 (15.4)	2 (3.5)
Private clinic/hospital	20 (18.3)	9 (17.3)	11 (19.3)
Other	1 (0.9)	1 (1.9)	0
TB test methodology			
Microscopy	41 (37.6)	15 (28.8)	26 (45.6)
Xpert MTB/RIF	68 (62.4)	37 (71.2)	31 (54.4)
Microscopy result (if test with microscopy)^b^			
1-9 (Scanty)	1 (2.6)	0	1 (4.0)
1+	10 (25.6)	5 (35.7)	5 (20.0)
2+	12 (30.8)	7 (50.0)	5 (20.0)
3+	16 (41.0)	2 (14.3)	14 (56.0)
Completed treatment (If ever treated for TB)			
Yes	11 (100)	6 (100)	5 (100)
No	0	0	0

Abbreviations: MTB/RIF, mycobacterium tuberculosis/resistance to rifampicin; TB, tuberculosis.

^a^ 1 missing value.

^b^ 2 missing values.

### Health-Related Quality of Life

Within the overall sample, none of the study participants had severe or extreme problems in any of the 5 dimensions of the EQ-5D-5L (Table 2). The highest percentage of participants had no problems (level 1) in the EQ-5D-5L dimensions of mobility (86 [78.9%]), self-care (88 [80.7%]), usual activities (67 [61.5%]), pain or discomfort (66 [60.6%]), and anxiety or depression (69 [63.3%]). The highest proportion of problems in the EQ-5D-5L dimensions was in pain or discomfort (43 [39.4%]) and the lowest was in self-care (21 [19.3%]).

**Table 2.** EQ-5D-5L Frequencies and Proportions Reported by Dimension and Level

Levels^a^	Participants, No. (%) (N = 109)				
	Mobility	Self-care	Usual activities	Pain/discomfort	Anxiety/depression
No problems	86 (78.9)	88 (80.7)	67 (61.5)	66 (60.6)	69 (63.3)
Slight problems	23 (21.1)	19 (17.4)	40 (36.7)	40 (36.7)	40 (36.7)
Moderate problems	0	2 (1.8)	2 (1.8)	3 (2.8)	0
Severe problems	0	0	0	0	0
Extreme problems	0	0	0	0	0

^a^ Descriptions for quality of life problems correspond to the EQ-5D-5L scale, which categorizes QoL problems as levels from 1 to 5.

EQ-5D-5L scores were significantly different between the 2 arms, which favored the intervention arm across all 5 dimensions (Table 3). The proportion without problems in the EQ-5D-5L dimensions (level 1) was higher among the intervention arm compared with the control arm (eg, pain or discomfort: 46 of 52 participants [88.5%] vs 20 of 56 [35.1%]; *P* < .001) and the proportion of participants with problems was higher among the control arm (eg, level 2 anxiety or depression: 32 of 56 [56.1%] vs 8 of 52 [15.4%]; *P* < .001).

**Table 3.** EQ-5D-5L Response Distribution by Study Arm

Dimensions^a^	Participants, No. (%)		*P* value
	Control (n = 56)	Intervention (n = 52)	
**Mobility**			
No problems	35 (61.4)	51 (98.1)	<.001^b^
Slight problems	22 (38.6)	1(1.9)	
Moderate problems	0	0	
Severe problems	0	0	
Extreme problems	0	0	
**Self-care**			
No problems	38 (66.7)	50 (96.2)	<.001^c^
Slight problems	17 (29.8)	2 (3.8)	
Moderate problems	2 (3.5)	0	
Severe problems	0	0	
Extreme problems	0	0	
**Usual activities**			
No problems	22 (38.6)	45 (86.5)	<.001^c^
Slight problems	33 (57.9)	7 (13.5)	
Moderate problems	2 (3.5)	0	
Severe problems	0	0	
Extreme problems	0	0	
**Pain/discomfort**			
No problems	20 (35.1)	46 (88.5)	<.001^c^
Slight problems	34 (59.6)	6 (11.5)	
Moderate problems	3 (5.3)	0	
Severe problems	0	0	
Extreme problems	0	0	
**Anxiety/depression**			
No problems	25 (43.9)	44 (84.6)	<.001^b^
Slight problems	32 (56.1)	8 (15.4)	
Moderate problems	0	0	
Severe problems	0	0	
Extreme problems	0	0	

Abbreviation: EQ-5D-5L, EuroQoL 5-dimension 5-level.

^a^ Descriptions for quality-of-life problems correspond to the EQ-5D-5L scale, which categorizes QoL problems as levels from 1 to 5.

^b^ *P* value from χ^2^ test.

^c^ *P* values from Fisher exact test.

Overall, 39 of 109 participants (35.8%) had full health (ie, a health state of 11111 based on EQ-5D-5L value sets); of whom, 36 of 39 (92.3%) were in the intervention arm. Comparing by arm, 3 of 57 (5.3%) of the control vs 36 of 52 (69.0%) of the intervention arms had full health.

The index value for the EQ-5D-5L ranged from 0.832 for health state 22322 to 1 for health state 11111. The maximum index value following full health was 0.976 for the health state 12111. The overall median (IQR) index value of the study participants was 0.964 (0.907-1). The minimum and maximum index values for the control arm were 0.832 for health state 22322 and 1 for health state 11111, respectively. For the intervention arm, these were 0.906 for health state 11222 and 1 for health state 11111, respectively. The EQ-5D-5L median (IQR) index value was significantly higher in the intervention arm (1 [0.974-1]) compared with the control arm (.908 [0.891-0.964]) (*P* < .001).

A log-binomial model was applied to determine the effect of sociodemographic, behavioral, and health characteristics of participants on HRQoL. In the simple regression model with a threshold of *P* < .20, 6 variables, namely trial arm, age, residency, smoking, khat (a stimulant) use, and previous TB treatment were potential candidates. In the multiple regression analysis, the trial arm was significantly associated with lower HRQoL. The risk of having lower HRQoL in the control arm was 1.49 times (95% CI, 1.35-1.65; *P* < .001) more likely compared with the intervention arm (eTable in Supplement 2).

### Patient Costs

The minimum and maximum overall TB treatment costs in the control arm were zero (for those treated in nearby facilities) and Ethiopian birr ETB 16 200 (US $310.34). For the intervention arm, these were zero and ETB 460 (US $8.81), respectively. Patients’ overall median postdiagnosis cost was ETB 80 (ETB 16-ETB 480) (US $1.53). The median (IQR) overall TB treatment cost was significantly higher in the control arm (ETB 432 [ETB 210-ETB 1980]) compared with the intervention arm (ETB 24 [ETB 16-ETB 48]). There was a statistically significant difference in the overall TB treatment cost between the 2 arms. The median (IQR) overall TB treatment cost in the control arm, assuming that they followed the procedure in the intervention arm, would have been ETB 32 (ETB 14-ETB 164). This means that if participants in the control arm had followed the intervention procedure, they would have saved a median 92.6% (93.3%-91.7%) (ETB 32 [ETB 14-ETB 164] vs ETB 432 [ETB 210-ETB 1980]) of their actual expenditure, with an estimated median saving of ETB 336 (ETB 156-ETB 1339) (*P* < .001).

Of the 109 total participants, 42 (38.5%; 95% CI, 29.4%-48.3%) faced catastrophic costs due to TB treatment, with a significantly higher proportion in the control arm (31 participants [54.4%; 95% CI, 40.7%- 67.6%]) compared with the intervention arm (11 participants [21.2%; 95% CI, 11.1%-34.7%]) (*P* < .001). None of the study participants reported using coping mechanisms. There was a statistically significant association between catastrophic cost and the study arm.

A log-binomial model was applied to identify risk factors of catastrophic cost. In multiple regression analysis, trial arm (adjusted risk ratio [ARR], 2.55; 95% CI, 1.58-4.13; *P* < .001), occupation (ARR, 2.58; 95% CI, 1.68-3.97; *P* < .001), number of cohabitants (ARR, 0.64; 95% CI, 0.43-0.95; *P* = .03), and smoking (ARR, 2.71; 95% CI, 1.01-7.28; *P* = .048) were statistically significant factors for patients facing catastrophic costs (Table 4). Thus, the risk of having catastrophic costs in the control arm was 2.55 times (ARR, 2.55; 95% CI, 1.58-4.13; *P* < .001) more likely compared with the intervention arm.

**Table 4.** Factors Affecting Catastrophic Costs

Variables	Participants, No.^a^	CRR (95% CI)	*P* value	ARR (95% CI)	*P* value
Arm					
Control	56	2.57 (1.45-4.57)	.001^b^	2.55 (1.58-4.13)	.001^c^
Intervention	52	1 [Reference]		1 [Reference]	
Sociodemographic characteristics					
Sex					
Women	37	2.11 (1.34-3.33)	.001^b^	1.16 (0.77-1.75)	.47
Men	71	1 [Reference]		1 [Reference]	
Age^d^		1.00 (0.98-1.03)	.68	NA	NA
Marital status					
Married	50	0.96 (0.59-1.54)	.86	NA	NA
Unmarried	58	1 [Reference]		NA	
Occupation					
No job	35	3.07 (1.93-4.89)	<.001^b^	2.58 (1.68-3.97)	.001^c^
Have job	73	1 [Reference]		1 [Reference]	
Education					
Below preparatory	77	1.14 (0.66-1.96)	.65	NA	NA
Preparatory and above	31	1 [Reference]		NA	
No. of cohabitants					
≤3	62	0.56 (0.35-0.89)	.02^b^	0.64 (0.43-0.95)	.03^c^
≥4	46	1 [Reference]		1 [Reference]	
No. of bedrooms					
1	67	0.77 (0.45-1.30)	.21	NA	NA
≥2	41	1 [Reference]		NA	
Household head					
No	47	1.29 (0.81-2.08)	.28	NA	NA
Yes	61	1 [Reference]		NA	
Residency					
Permanent	88	1.36 (0.67-2.79)	.40	NA	NA
Temporary	20	1 [Reference]		1 [Reference]	
Behavioral characteristics					
Smoking per day					
Never	90	2.60 (0.90-7.50)	.08^b^	2.71 (1.01-7.28)	.048^c^
Yes	18	1 [Reference]		1 [Reference]	
Khat					
Never	87	1.79 (0.80-3.99)	.16^b^	0.94 (0.40-2.17)	.88
Yes	21	1 [Reference]		1 [Reference]	
Alcohol					
Never	67	1.37 (0.81-2.31)	.25	NA	NA
Yes	41	1 [Reference]		NA	
Disease conditions					
HIV					
Negative	93	0.97 (0.49-1.89)	.92	NA	NA
Positive	15	1 [Reference]		NA	
TB treatment					
New	98	4.18 (0.64-27.25)	.13^b^	2.66 (0.61-11.52)	.19
Relapse	10	1 [Reference]		1 [Reference]	

Abbreviations: ARR, adjusted relative risk; CRR, crude relative risk; NA, not applicable; TB, tuberculosis.

^a^ Total of 108 participants as 1 excluded for having HIV infection.

^b^ *P* value from χ^2^ test.

^c^ *P* values from Fisher exact test.

^d^ Reference was per year difference.

## Discussion

To our knowledge, this is the first randomized clinical trial investigating the effects of MERM-enabled self-administered therapy on HRQoL and costs for patients with TB compared with the standard DOT. Consistent with our hypothesis, patients with TB who received a 15-day TB medication supply in the MERM device to self-administer and return every 15 days (intervention arm) had a significantly higher HRQoL and lower catastrophic and total costs compared with those who visited health care facilities each day to swallow their daily dose with direct observation by clinicians (control arm).

This study provides strong evidence of the HRQoL benefits of MERM in adults with new or previously treated, bacteriologically confirmed, drug-sensitive pulmonary TB who were eligible to start first-line anti-TB therapy. The EQ-5D-5L median index value was significantly higher in the intervention arm compared with the control arm, and the EQ-5D-5L health state utility value was higher for the intervention arm compared with the control arm, with patients in the control arm being more likely to have a lower HRQoL (ie, at least 1 health problem). The risk for lower HRQoL was 49% likely higher in DOT control arm compared with the MERM intervention arm. This indicates that this intervention has the potential to maintain the economic, psychological, and social well-being of patients with TB as compared with the standard DOT. The main explanation is that, unlike in-person daily DOT, patients who used the MERM device visited the health care facility every 15 days and this significantly reduced vulnerability of patients to the underlying barriers including costs for travel, food, and accommodation for daily in-person DOT. Studies demonstrate that quality of life is an important indicator of the effectiveness of a TB treatment strategy.^24,25^ TB is not simply confined to the sphere of biomedicine, but also physical, mental, and social sufferings that the current TB treatment strategy exacerbates.^26,27,28^ Daily in-person DOT is reported by patients as a tiresome and demanding procedure,^29,30,31,32^ and the problem is severe in challenging conditions that impair population mobility, such as the onset of the COVID-19 pandemic.^33,34,35^

In this study, the overall EQ-5D-5L median index value was equivalent to a previous score for patients with HIV in Ethiopia.^36^ None of the study participants had severe or extreme problems in any of the 5 dimensions. The highest proportion of problems in the EQ-5D-5L dimensions was in pain or discomfort and the lowest was in self-care. In the modified regression done to determine the effect of sociodemographic, behavioral, and health characteristics of participants on HRQoL, only the trial arm was found to be a statistically significant factor.

In this study, the cost of treatment was measured to assess how TB may affect the welfare of households and individuals by estimating the costs that patients with TB and their households incur during treatment, with a focus on costs related to anti-TB drug pick-up, guardian costs, and coping costs over the 2-month intensive phase. The mean monthly income of study participants was US $67.50, which was lower than the national average of US $183.50.^37^ The median cost was significantly lower in the intervention arm compared with the control arm. If patients in the control arm followed the intervention procedure, they would have the possibility of saving about 92.6% of their current expenditure on TB treatment. Previous studies done in different countries reported a lower patient cost of TB treatment with the use of digital technology–observed therapy compared with DOT under various cost consequences,^38,39,40,41^ which is in agreement with the current study, while none of the previous studies reported on cost-effectiveness relevant to a MERM device.

Similarly, a significantly lower proportion of patients with TB in the intervention arm faced catastrophic costs as compared with those in the control arm at a 20% cut-off point. Trial arm, occupation, number of cohabitants, and smoking cigarettes were the most important factors in estimating catastrophic cost. The financial burden of treatment remains a critical issue for patients with TB preventing them from being retained in care,^42,43,44^ which alternative mechanisms including the use of digital health may alleviate.

### Limitations

The present study had some limitations. The overall sample size was modest. This study was carried out in Addis Ababa, Ethiopia, and this limits the generalizability of our results. The WHO’s tool to estimate patient costs had the potential for recall bias and omits costs related to other types of TB-related morbidity, such as pain and nausea medications. The EQ-5D-5L was completed once after treatment using a cross-sectional design and thus did not show pre-post treatment changes in participant health status. Despite these limitations, this study clearly compared the effects of MERM-observed self-administered therapy on HRQoL and costs for patients with TB vs the standard DOT in Ethiopia, one of the countries with the highest burden of TB but poorly represented in such clinical trials.

## Conclusions

In this study of patients with TB, MERM device-observed self-administered therapy was associated with higher HRQoL and lower catastrophic costs compared with the standard DOT. Patient-centered digital health technologies could have the potential overcoming structural barriers to anti-TB therapy.

## Notes

Supplement 1.
Trial Protocol

Supplement 2. **eTable.** Demographic, behavioral, and clinical factors and risk of low health-related quality of life (n=108)

Supplement 3.
Data Sharing Statement
