# Supplementary material for: Effect of Digital Medication Event Reminder and Monitor-Observed Therapy vs Standard Directly Observed Therapy on Health-Related Quality of Life and Catastrophic Costs in Patients With Tuberculosis: A Secondary Analysis of a Randomized Clinical Trial
Source: JAMA Netw Open. 2022 Sep 15;5(9):e2230509. doi: 10.1001/jamanetworkopen.2022.30509 (PMC9478770; doi:10.1001/jamanetworkopen.2022.30509)
Supplement: Supplement 1. — Trial Protocol [file jamanetwopen-e2230509-s001.pdf]

Manyazewal T, Woldeamanuel Y, Fekadu A, Holland DP, Marconi VC. Effect of digital medication event reminder and monitor-observed therapy vs standard directly observed therapy on health-related quality-of-life and catastrophic costs in patients with tuberculosis: a secondary analysis of a randomized clinical trial. *JAMA Netw Open.* 2022;5(9):e2230509. doi:10.1001/jamanetworkopen.2022.30509

Electronic pillbox-enabled self-administered therapy versus standard directly observed therapy for tuberculosis medication adherence and treatment outcomes in Ethiopia: a multicenter randomized controlled trial (SELFTB trial)

Principal Investigator:

Dr. Tsegahun Manyazewal. tsegahunm@gmail.com, tsegahun.manyazewal@aau.edu.et
Addis Ababa University, College of Health Sciences, Center for Innovative Drug Development and Therapeutic Trials for Africa (CDT-Africa). Tel: (+251)1118787311, Fax: (+251) 115511079, P.O.Box 9086 Addis Ababa, Ethiopia

Co-investigators:

Prof. Vincent C. Marconi. vcmarco@emory.edu
Emory University School of Medicine and Rollins School of Public Health, Atlanta, GA, 30322, USA.

Dr. Yimtubezinash Woldeamanuel
yimtubezenash.wamanuel@aau.edu.et
Addis Ababa University, College of Health Sciences. Tel: (+251)1118787311, Fax: (+251) 115511079, P.O.Box 9086 Addis Ababa, Ethiopia

Dr. David P Holland. david.holland@emory.edu
Emory University School of Medicine and Rollins School of Public Health, Atlanta, GA, 30322, USA.

Prof. Abebaw Fekadu. abebaw.fekadu@aau.edu.et
Addis Ababa University, College of Health Sciences, Center for Innovative Drug Development and Therapeutic Trials for Africa (CDT-Africa). Tel: (+251)1118787311, Fax: (+251) 115511079, P.O.Box 9086 Addis Ababa, Ethiopia.

Prof. Henry M. Blumberg: hblumbe@emory.edu
Emory University School of Medicine and Rollins School of Public Health, Atlanta, GA, 30322, USA.

Collaborating Institutions:

Addis Ababa University, Addis Ababa, Ethiopia
Emory University School of Medicine, Atlanta, Georgia, 30322, USA
Armauer Hansen Research Institute, Ethiopia
Fogarty International Center of the National Institute of Health, USA

CONTENTS

1. PROTOCOL SUMMARY 1
   1.1. Synopsis 1
   1.2. Scheme 4
2. BACKGROUND 5
   2.1. Study rationale 5
   2.2. TB Digital Adherence Technologies: evidence in literature 9
3. CONCEPTUAL FRAMEWORK 11
4. HYPOTHESIS 12
5. OBJECTIVES 13
   5.1. Primary objectives 13
      5.1.1. Treatment adherence 13
      5.1.2. Treatment outcomes 13
   5.2. Secondary objectives 13
      5.2.1. HRQoL and catastrophic cost 13
      5.2.2. Usability and treatment satisfaction 13
6. OUTCOME MEASURES 13
   6.1. Primary outcome 13
      6.1.1. Level of adherence 13
      6.1.2. Sputum conversion 14
   6.2. Secondary outcomes 14
      6.2.1. Negative IsoScreen urine isoniazid test 14
      6.2.2. Adverse treatment outcome 14

6.2.3. Self-reported adherence ... 14
6.2.4. Health-related quality of life (HRQoL) ... 14
6.2.5. Catastrophic costs ... 14
6.2.6. Post-diagnostic cost from an individual patient's perspective ... 14
6.2.7. Patient-reported treatment satisfaction ... 14
6.2.8. Patient-reported usability of the MERM device ... 15

7. SIGNIFICANCE AND TRANSFORMING POTENTIAL ... 15

8. METHODS ... 16

8.1. Materials ... 16

8.2. Trial design ... 18

8.3. Setting ... 18

8.4. Site selection ... 19

8.5. Study participants ... 20

8.5.1. Study participants (TB patients) ... 20
8.5.1.1. Study population ... 20
8.5.1.2. Inclusion criteria ... 20
8.5.1.3. Exclusion criteria ... 20
8.5.1.4. Justification for inclusion and exclusion ... 21
8.5.1.5. Sampling and sample size ... 21
8.5.2. Provider participants ... 21
8.5.2.1. Study population ... 21
8.5.2.2. Inclusion criteria ... 22
8.5.2.3. Exclusion criteria ... 22
8.5.2.4. Justification for inclusion and exclusion ... 22
8.5.2.5. Sampling and sample size ... 22

8.6. Interventions ... 22

8.7. Study procedure ... 23

8.8. Definition of terms ... 26

8.8.1. Medication adherence ... 26
8.8.2. Effectiveness ... 26
8.8.3. Cost-effectiveness ... 26

8.8.4. Usability 26
8.8.5. TB patient 27
8.8.6. TB patient cost 27
8.8.7. New cases 27
8.8.8. Bacteriologically confirmed TB case 27
8.8.9. Loss to follow-up 27
8.8.10. Treatment completed 27
8.8.11. Healthcare facility 27
8.9. Data management 27

9. STATISTICAL ANALYSIS 28

10. QUALITY ASSURANCE 29

11. STUDY ASSESSMENT AND DISCONTINUATION 29

11.1. Participant discontinuation 29

11.2. Unexpected or adverse events 30

11.3. Interim monitoring and analysis 30

12. ETHICAL CONSIDERATIONS 31

12.1. Statement of compliance 31

12.2. Ethical review 31

12.3. Informed consent 32

12.4. Confidentiality 32

12.5. Payment for participation 32

13. REFERENCES 33

1. PROTOCOL SUMMARY

1.1. Synopsis

Title: Electronic pillbox-enabled self-administered therapy versus standard directly observed therapy for tuberculosis medication adherence and treatment outcomes in Ethiopia: a multicenter randomized controlled trial (SELFTB trial)

Hypothesis: The use of a digital medication event reminder and monitor device-observed self-administered therapy provides non-inferior medication adherence and treatment outcomes for patients with TB compared with the standard in-person directly-observed therapy (DOT) in Ethiopia, one of the low-income countries with the highest burden of TB.

Objectives: Primary objectives:

1. To assess whether a digital medication event reminder monitor (MERM) device-observed self-administered therapy improves adherence in patients with TB compared with the standard DOT.
2. To assess whether MERM-observed self-administered therapy improves treatment outcomes in patients with TB compared with the standard DOT.

Secondary objectives:

3. To evaluate if MERM-observed therapy provides higher health-related quality-of-life (HRQoL) and lower catastrophic costs compared to the standard DOT
4. To evaluate the patient-reported usability and treatment satisfaction with MERM-observed self-administered TB therapy compared with the standard in-person DOT.

Outcome measures: Primary outcomes:

1. Level of adherence: Individual-level percentage adherence over the two-month intensive phase measured by adherence records compiled from MERM device vs. DOT records.

2. Sputum conversion: Participants with sputum smear converted following the standard two-month intensive phase treatment.

Secondary outcomes

3. Negative IsoScreen urine isoniazid test: Participants having at least one negative urine isoniazid test result (IsoScreen test, GFC Diagnostics Ltd, Bicester, England)
4. Adverse treatment outcome: Participants having at least one of the three

events: treatment not completed; death; or loss to follow-up

5. Self-reported adherence: Participants who self-reported to have forgotten to take their medication

6. Health-related quality of life (HRQoL): The association between MERM-observed therapy and HRQoL, with the HRQoL measured and calculated for each participant by arm using the EuroQoL 5-dimension 5-level (EQ-5D-5L) score ranging from 0 to 1, with a higher score designating better HRQoL.

7. Catastrophic costs: Participants with overall TB treatment cost exceeding or equivalent to 20% of their income.

8. Post-diagnostic cost from an individual patient's perspective: Participant's cumulative direct costs (out-of-pocket costs related to anti-TB drug pick-up) and indirect costs (guardian and coping costs) over the two-month intensive phase.

9. Patient-reported treatment satisfaction: Participant's treatment satisfaction measured using the treatment satisfaction questionnaire for medication version 1.4 (TSQM v1.4) tool on a scale from 0 to 100, with a higher score indicating better satisfaction.

10. Patient-reported usability of the MERM device: Participant’s experience using the MERM device measured by an 18-item questionnaire and the score transformed into a scale from 0 to 100, with a higher score indicating better usability (Intervention arm only).

Methods: *Design*: A multicenter, randomized, controlled, open-label, non-inferiority, effectiveness-implementation type 2 hybrid trial in ten healthcare facilities in Addis Ababa, Ethiopia. The study will not dictate diagnosis and treatment for TB; thus, it will not introduce or use new medications.

Setting: The study country is Ethiopia – a high TB-burden, low-income country located in sub-Saharan Africa. In Addis Ababa, a total of 94 public health centers provide TB care and treatment services under the DOT program. This study stratifies the 94 health centers on the bases of the 10 sub-cities where they are located. From each stratified group, one health center with the largest TB client load will be selected, with a total of 10 health centers to be included. Health management information system (HMIS) quarterly data (April 01 - June 30, 2019) taken from the Addis Ababa Regional Health Bureau will be used for the purpose.

Participants: The source population will be all new patients with TB symptoms who come to a study site and undergo bacteriological screening during the study period or TB patients bacteriologically confirmed elsewhere and referred to a study site for TB treatment. Inclusion criteria: i) patients with new, or previously treated, bacteriologically-confirmed drug-sensitive pulmonary TB, ii) eligible to start the

standard 6-month first-line anti-TB medication, iii) outpatient status at the time of screening and enrollment, iv) men or women age >=18 years, and v) able and willing to provide informed consent. The sample size calculation yields a sample of 57 in each arm for a total of 114 participants.

Intervention: Participants will be randomly assigned (1:1) to receive a 15-day TB medication supply in the evriMED500® MERM device to self-administer and return every 15 days (intervention arm) or the standard in-person DOT (control arm). Both will be followed throughout the standard two-month intensive treatment phase (2RHZE). The MERM device has an electronic module and a medication container that records adherence, stores medication, emits audible and visual on-board alarms to remind patients to take their medications on time and refill, and enables providers to download the data and monitor adherence.

Study Duration: The study duration is 12 months from opening enrollment until completion of data analyses. Participant duration is 2 months for each individual participant to complete all visits.

1.2. Scheme

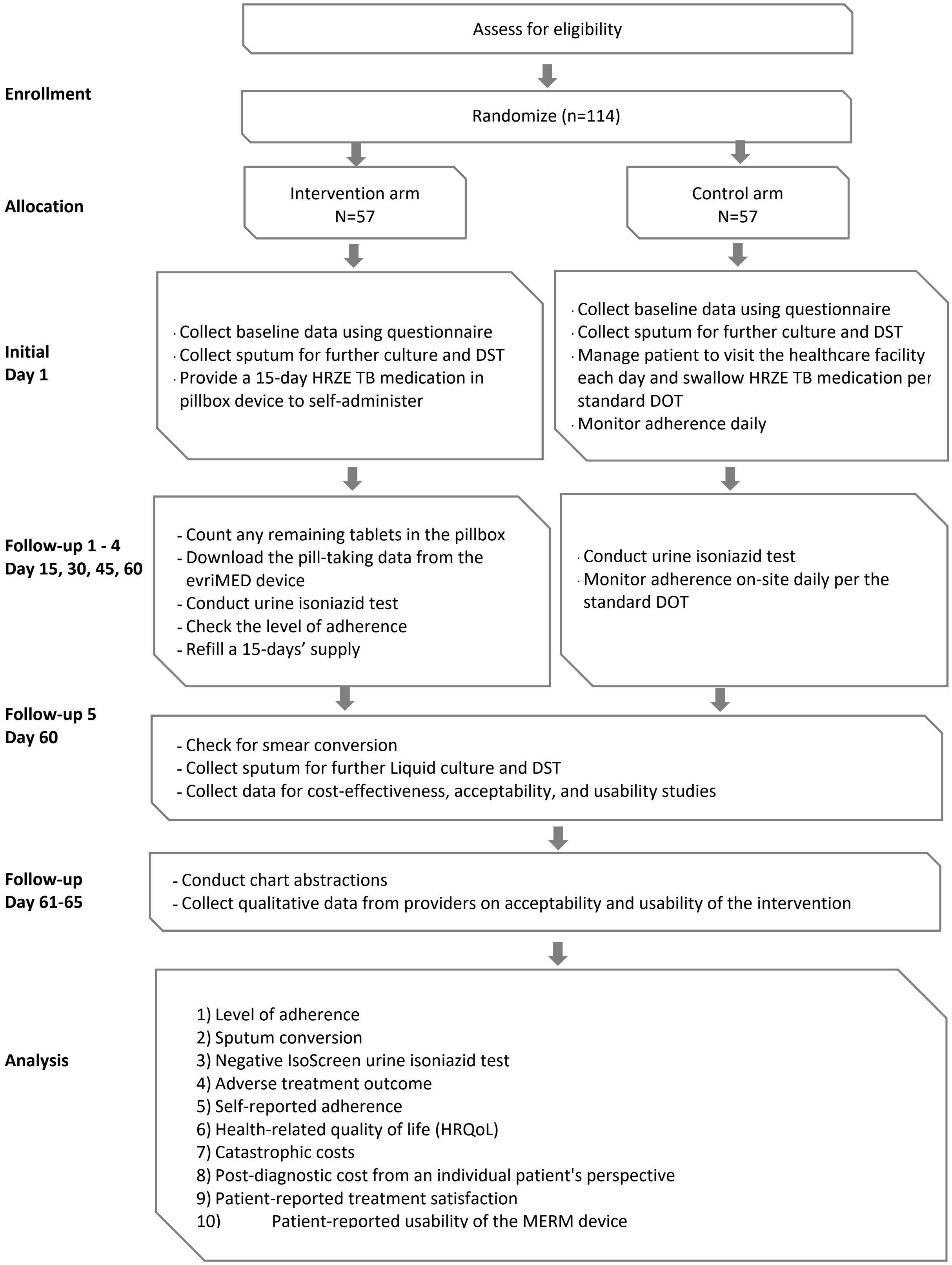

Figure 1*: Schematic diagram of the study*

2. BACKGROUND

2.1. Study rationale

The World Health Organization (WHO) revealed its commitment to tuberculosis (TB) patients in its End TB strategy that "everyone with TB should have access to the innovative tools and services they need for rapid diagnosis, treatment, and care; this is a matter of social justice, fundamental to our goal of universal health coverage". This commitment is a collective responsibility towards human rights; making sure that no family is burdened with avoidable death or catastrophic expenses due to TB by 2030. However, taking pride in the slogan "End TB by 2030" is insufficient for true progress. In order to translate aspirational goals into reality, practical solutions should be sought. The strategy might appear to be a zero-sum if interventions on the disease neglect key socioeconomic burdens that individual patients are incapable of avoiding. The association between TB and poverty is a reality (1-5), but the disease is a global health security threat that urgently needs collective resources for mutual welfare (6,7). In the last three decades, different strategies and care packages have been formulated and implemented to halt the disease. However, improvements are not as expected (8,9); instead, a drug-resistant form of the disease is spreading [10-12], with globalization and migration fueling multiple strains of the disease worldwide [13-15]. The main debate here is how we can meet End TB's vision of "A world free of TB: zero deaths, disease and suffering due to TB" without bargaining the inherent values and dignity of TB patients. It remains unclear how low-income countries would be able to meet one of the four key indicators of the strategy "Zero TB-affected families facing catastrophic costs due to TB by 2035" in situations where management of TB treatment still relies on directly observed therapy (DOT).

TB patients from the poorer segments of society are not benefiting from care delivery innovations: a contradiction between "Global Commitment to End TB" and "reality on the ground". Management of their treatment still depends on DOT, where controversies are everywhere on its potential turning the End TB strategy to reality. DOT has been viewed as an efficient strategy for treatment adherence [16,17], while evidence has demonstrated that it poses an economic and social burden to TB patients living in low-income countries [18-25]. Treatment of TB lasts for at least six months, where patients in the intensive phase of DOT need to collect their medication at healthcare facilities daily and swallow tablets under the direct observation of a healthcare worker throughout the intensive phase. This process challenges not only patients but also the healthcare system as it is labor-intensive to supervise

the daily treatment of large numbers of TB patients [18,19,26], which portend a dystopia in the epidemiology of the disease.

There is no single measurement strategy deemed a universal solution to improve adherence; however, DOT is yet the only option for management of TB treatment adherence in low-income countries, despite multi-method approaches used in high-income countries. There is a natural tendency to focus on patient-related domains as core determinants of TB treatment adherence, while for other chronic diseases, treatment relies on patient self-management, giving them freedom and ownership of their own and their communities' health. Adherence is a multidimensional phenomenon determined by the interplay of five sets of factors, of which patient-related factors are just one determinant (Figure 2) [27,28]. The common belief that patients are solely responsible for taking their treatment is misleading and most often reflects a misunderstanding of how other factors affect people's behavior and capacity to adhere to their treatment. It also places the burden solely on the patient and stigmatizes and demoralizes the patient who is deemed delinquent or a defaulter when this breaks down.

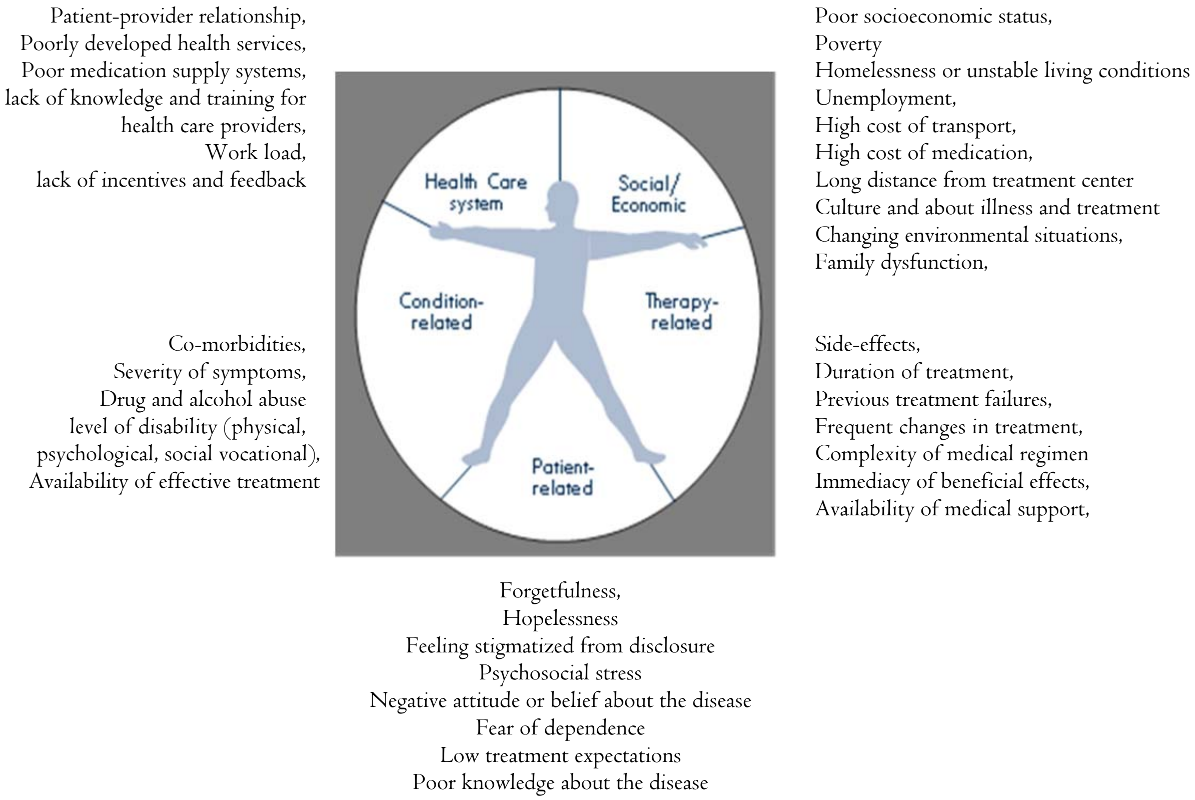

Figure 2: Five dimensions of adherence, adopted from (27, 28)

Ethiopia, where the proposed study will be conducted, is among countries highly encumbered by the TB epidemic, and one of the least resourced in the world. According to the 2018 global TB report, there were 117,705 TB cases reported in the country, and of the annual $93 million needed for TB care and control, the country's domestic contribution was only 11% [29]. Despite TB care and treatment services being delivered free of charge, TB patients are facing out-of-pocket payments [30,31] and income losses [32] due to transportation, accommodation, and food to get treatment at healthcare facilities: tackling their adherence to treatment and forcing them to stop working, sell their properties, borrow money and reduce their overall income [33]. These, in turn, are increasing rates of loss to follow-up, disease relapse, and drug resistance [34]. The spread of TB due to patients traveling daily to healthcare facilities for medication is increasing the transmission potential of the disease, especially in the capital city Addis Ababa, where mobility is making the city crowded - as manifested through housing conditions and the public transport. Different studies conducted in Addis Ababa reported that TB patients consider their daily DOT visits as pointless [35-39], and providers see DOT as a very challenging strategy for TB patients (37). As a result, daily DOT survives in principle, while implementation is irregular as both TB patients and providers have uncertainties with it. Providers witness that TB patients prefer taking the tablets at home once they have the necessary advice and counseling [35,37]. Patients complain that they travel for daily DOT on foot under exhaustive road conditions for up to two hours and 2.5 kilometer, taking several rests on their way because of their sickness (35,38,40). They spend much on transportation [37,41] and some of them get fired from their job as they were absent for the daily DOT (35,39,40). Providers witness how patients are seen when they exhaust (35), and patients took their daily DOT with dissatisfaction [39]. Some patients claim that once they are informed of the disease on day one, no one talks to them the following days and they just swallow the drug and go back (37). Besides, they face stigma on their way to daily DOT, and they change their name in the TB clinic for they do not want to be notified as TB patients [41]. The challenge is similar in other parts of Ethiopia (42-45). According to the WHO 2018 report [29], TB treatment coverage in the country is 68%, which is minimum even to accommodate the current DOT need. The estimated percentage of MDR/RR TB cases is significantly higher in previously treated cases [14% (6.7–25)] than new cases [2.7% (1.6–4.1)]; which indicates that management of TB treatment under DOT is problematic in the country. There were 680 MDR/RR-TB and four XDR-TB laboratory-confirmed cases in the country in 2017 (29), while many more were left undiagnosed due to limited availability of the service.

The Ethiopian national TB program adheres to the WHO End TB strategy for its TB functions. The national comprehensive TB guideline revised in 2016 (46) standardize the provision of one first-line anti-TB treatment regimen for both new and previously treated TB patients. The treatment is administered in two phases (Figure 3). The initial (intensive) phase consists of a fixed-dose combination of four drugs [(isoniazid (H), rifampicin (R), pyrazinamide (Z) and ethambutol (E), with 75/150/400/275 mg strength] to be taken for the first eight weeks for new cases. The second (continuation) phase consists of a fixed-dose combination (RH, with 75/150 mg strength) to be taken for four months for new cases, which may be extended to 10 months if the disease involves the central nervous system, bones or osteoarticular spaces. Management of treatment is entirely driven by DOT implementation principles and modalities.

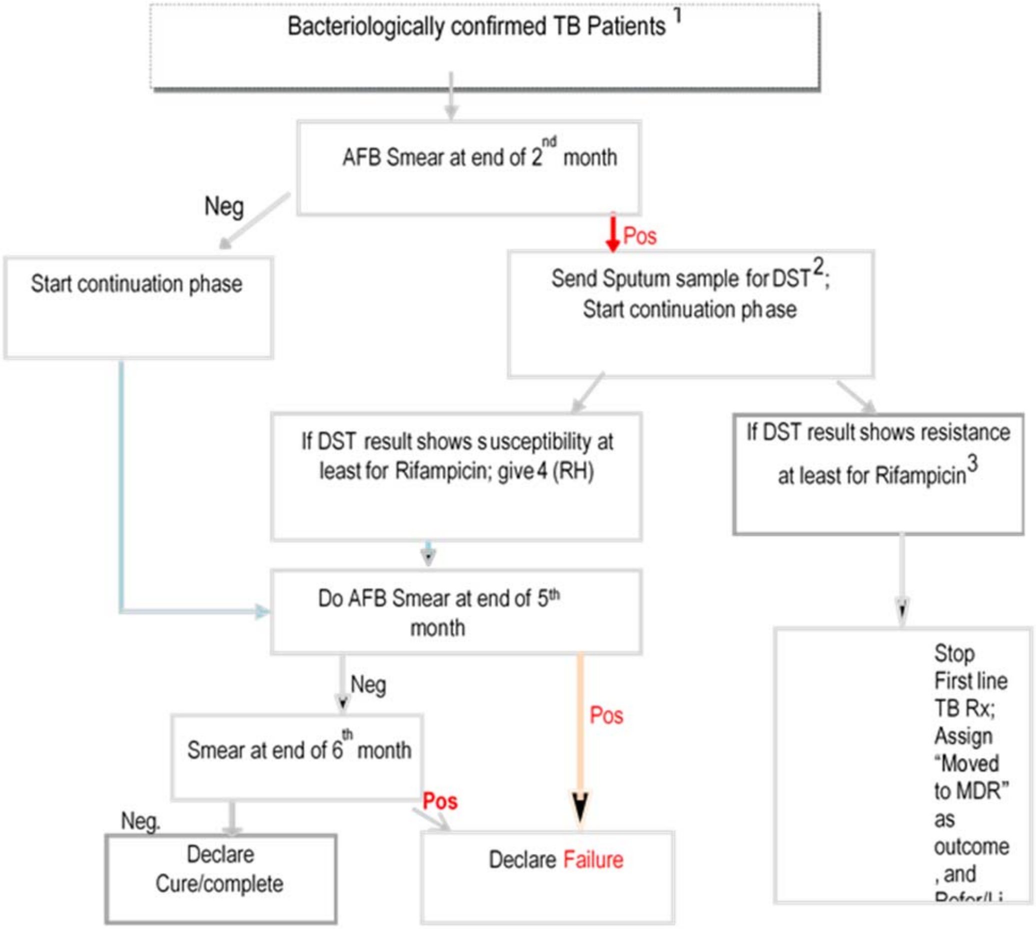

[1]Bacteriolgically confirmed TB patients include those diagnosed by a positive result on AFB microscopy, Xpert MTB/RIF Assay or culture;[2]DST may be performed from one sputum sample using Xpert MTB/RIF, LPA or conventional DST based on availability. Information on rifampicin may be enough to decide on Next Action.[3] if DST result shows resistance to INH but susceptible to Rifampicin; treat with RHZE for 9 months.

Figure 3. Flow Chart for Sputum AFB monitoring for bacteriologically confirmed PTB Patients (46)

In Ethiopia, there have been no alternative TB treatment strategies to DOT to address patient-specific adherence barriers or challenges. This would have a significant impact on the End TB indicator of "Zero TB-affected families facing catastrophic costs due to TB by 2035". Besides, Multidrug-resistant TB (MDR-TB) remains a serious issue in the country, with a high burden [47] and transmission potential [48]. Such evidence discloses the difficulty of Ethiopia to meeting the overall End TB strategy targets by 2035 [49] unless significant investment is made to shape the current DOT strategy to a more advanced, technology-led, patient-centered strategy that could be responsive to individual patient preferences, needs and values.

The challenge with daily DOT is across sub-Saharan African countries, where DOT did not provide a significant solution to poor treatment adherence; instead, home and community-based therapies were shown to be possible alternative strategies to health facility DOT as studies held in Tanzania (50-52), Kenya (53,54), Zambia (55), South Africa (56,57), and Eretria (58) reported. The WHO 2018 Global TB Report (29) showed that TB remains a leading cause of death in Africa. The continent accounts for a quarter of new TB cases and TB deaths worldwide, with 2.5 million people falling ill and 417,000 people dying from TB annually. Of the total TB patients co-infected with HIV globally, 72% of them live in Africa (29). The true burden of DR-TB in the continent is poorly described, with only 51% of countries having formal data in the WHO global TB database, where DR-TB is largely missed and this requires a major effort to achieve the 2035 targets (59).

2.2. TB Digital Adherence Technologies: evidence in literature

DOT-dependent treatment management alone has shown to be insufficient to ensure medication adherence and treatment outcomes (60). Understanding this challenge, the global scientific community has invented Digital Adherence Technologies (DATs) that has transformed DOT to SAT. Such technologies, in general, have been shown to improve TB treatment outcomes [61-64] and substantially save costs [65], while available data are yet limited for better conclusions of their effectiveness in different countries and settings [66-69]. Assessing the TB DATs landscape, and understanding the barriers of non-adherence in its End TB Strategy, the WHO has endorsed in 2017 three DATs: short message service/mobile phone texting (SMS), Medication Event Reminder Monitor (MERM), and video-supported Directly Observed Therapy (VDOT) [70]. The SMS involves sending a standardized and understandable text message to the TB patient regularly to remind and motivate

them to take the prescribed medications. MERM involves the use of an electronic pillbox consisting of an automated pill container that emits audible and visual alerts to remind patients to take medication and transmits a signal to healthcare providers either when the box is opened or when it remains unopened for a given time and remotely monitor adherence. MEM pillboxes were in use several decades ago to monitor the usage of pill containers of different medications. MERM sleeves (99DOTS prototype) have an additional component that each medication blister is wrapped in 99DOTS envelopes to send to providers a hidden signal unrecognized by the patient. VDOT involves video communication between patients and healthcare providers, where providers watch patients take their medication, live or self-recorded, and provide advice and support. VDOT is mediated primarily through internet-enabled smartphones, and internet access is a critical component [70].

There have been limited studies conducted on DATs. For available studies, effectiveness at different resource-limited countries has been a subject of research and controversy. Table 1 summarizes the findings of recent studies conducted on the three DATs.

Table 1: summary of existing literature on TB digital adherence technologies

Reference	Country	Design	Outcome measure	Finding
**SMS**				
Bediang et al 2018 [71]	Cameroon	RCT: SMS vs DOT	Treatment success	No significant difference
Fang et al 2017 [72]	China	RCT: SMS vs DOT	Treatment completion rate	High
Mohammed et al 2016 [73]	Pakistan	RCT: SMS vs DOT	Treatment success	No significant difference
Liu et al 2015 [74]	China	RCT: 4 arms	Pill count, adherence	No significant improvement
Iribarren et al 2013 [75]	Argentina	Cross-sectional	feasibility and acceptability	acceptable and feasible
**MERM**				
Onwubiko et al 2019 [76]	USA	RCT: MERM vs DOT	Treatment completion	Low
Park et al 2019 [77]	Morocco	RCT: MERM vs DOT	Treatment success	High
Liu et al 2017 [78]	China	Multi-method	User performance, satisfaction	High
Broomhead et al 2012 [79]	USA	Cross-sectional	Treatment outcome, cost	High, lower cost per patient
Thakkar et al 2019 [80]	India	Cohort, 99DOTS used	Treatment adherence	High
**VDOT**				
Lam et al 2018 [81]	USA	RCT. VDOT vs DOT	Treatment completion	High
Garfein et al 2018 [82]	USA	RCT: VDOT vs DOT	Adherence, cost	High, lower cost
Nguyen et al 2017 [83]	Vietnam	Prospective cohort	Treatment adherence	High
Chuck et al 2016 [84]	USA	RCT: VDOT vs DOT	Treatment outcomes	No significant difference
Garfein et al 2015 [85]	USA, Mexico	Single-arm trial	Treatment adherence	High in both settings

RCT: randomized controlled trial; USA: United States of America

Considering the low-impact of SMS for TB treatment adherence and the high investment and technology need of VDOT, this study plans to focus on MERM (electronic pillbox) intervention. It is the most commonly used device to promote medication adherence for different diseases. With a pillbox, patients can self-manage their medications, identify whether they have taken the dose or not, and minimizes the rate of medication errors (28). Previous studies found that people who used a pillbox had better treatment adherence (86-88). This device is associated with improvements in medication adherence and, subsequently, with better health [86]. MERM electronic pillbox has an additional feature of reminding patients to take their drugs with visual and audio alerts, providing information about treatment adherence (89). However, there are concerning limitations with MERM, such as battery replacement or wireless signaling for sending and receiving messages, that are relevant to developing countries like Ethiopia and need further exploration.

3. CONCEPTUAL FRAMEWORK

Figure 4 illustrates the conceptual framework that provides the theoretical basis of the study and demonstrates potential outcome and long-term impact on it on End TB if certain conditions are fulfilled.

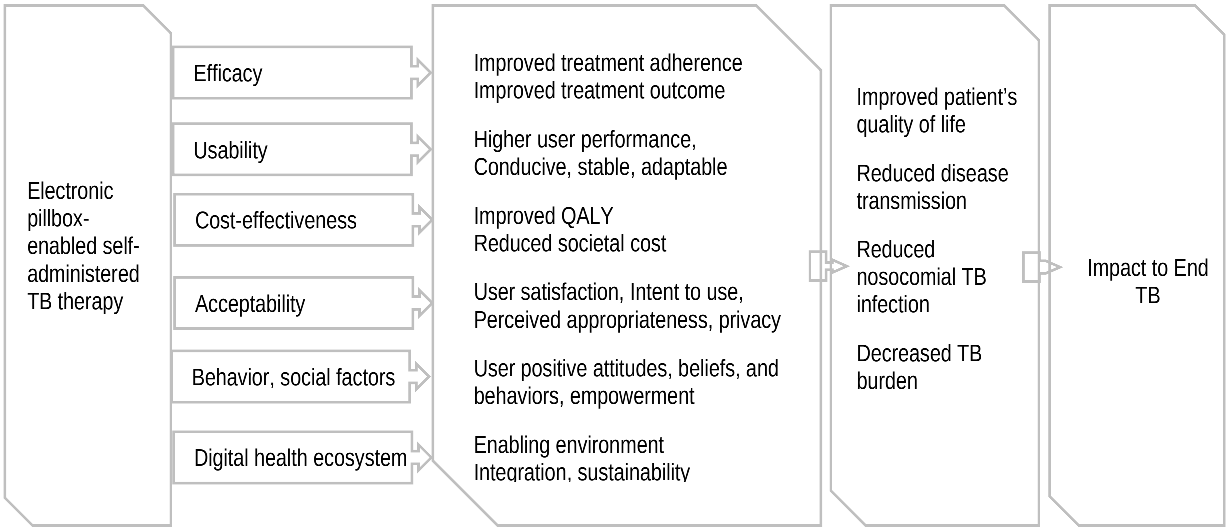

Figure 4:Conceptual framework of the study

This framework applies the functional linkage between TB innovation and the End TB strategy as a common ground that any TB intervention should have an ultimate goal realizing the End TB strategy, with patient rights "practically" guaranteed. This can be traced to the origins of "empowerment

theory” (90) - connecting individual-welling with the larger political and social environments through which individuals and groups gain greater control over their lives, acquire rights, and reduce marginalization.

The framework is guided by WHO’s recent (2017) handbook for the use of digital technologies to support tuberculosis medication adherence [70] and the WHO’s 2016 guideline for monitoring and evaluating digital health interventions [91]. The success of the MERM pillbox as a medication adherence device relies on its efficacy, usability, cost-effectiveness, acceptability, behavioral and social determinants of patients and the broader digital health ecosystem of the implementing country. If these fairly benefits the system, it is likely that TB treatment will succeed, the patient’s quality of life will be improved, disease transmission and nosocomial transmission will cease, and will reduce mortality and morbidity due to the disease, and ultimately the End TB strategy will be met.

4. HYPOTHESIS

- The use of a digital medication event reminder and monitor device-observed self-administered therapy provides a non-inferior medication adherence and treatment outcomes for patients with TB compared with the standard in-person DOT in Ethiopia, one of the low-income countries with the highest burden of TB

5. OBJECTIVES

5.1. Primary objectives

5.1.1. Treatment adherence

To assess whether a digital medication event reminder monitor (MERM) device-observed self-administered therapy improves adherence in patients with TB compared with the standard DOT.

5.1.2. Treatment outcomes

To assess whether MERM-observed self-administered therapy improves treatment outcomes in patients with TB compared with the standard DOT.

5.2. Secondary objectives

5.2.1. HRQoL and catastrophic cost

To evaluate if MERM-observed therapy provides higher HRQoL and lower catastrophic costs compared to the standard DOT

5.2.2. Usability and treatment satisfaction

To evaluate the patient-reported usability and treatment satisfaction with MERM-observed self-administered TB therapy compared with the standard in-person DOT

6. OUTCOME MEASURES

6.1. Primary outcome

6.1.1. Level of adherence

- Individual-level percentage adherence over the two-month intensive phase measured by adherence records compiled from MERM device vs. DOT records.

6.1.2. Sputum conversion

- Participant with sputum smear converted following the standard two-month intensive phase treatment.

6.2. Secondary outcomes

6.2.1. Negative IsoScreen urine isoniazid test

- Number of participants with negative IsoScreen urine isoniazid test.

6.2.2. Adverse treatment outcome

- Participants having at least one of the three events: treatment not completed; death; or loss to follow-up.

6.2.3. Self-reported adherence

- Participants who self-reported to have forgotten to take their medication.

6.2.4. Health-related quality of life (HRQoL)

- The association between MERM-observed therapy and HRQoL, with the HRQoL measured and calculated for each

6.2.5. Catastrophic costs

- Participants with overall TB treatment cost exceeding or equivalent to 20% of their income.

6.2.6. Post-diagnostic cost from an individual patient's perspective

- Participant's cumulative direct costs (out-of-pocket costs related to anti-TB drug pick-up) and indirect costs (guardianand coping costs) over the two-month intensive phase.

6.2.7. Patient-reported treatment satisfaction

- Participant's treatment satisfaction measured using the treatment satisfaction questionnaire for medication version 1.4 (TSQM v1.4) tool on a scale from 0 to 100, with a higher score indicating better satisfaction.

6.2.8. Patient-reported usability of the MERM device

- Participant's experience using the MERM device measured by an 18-item questionnaire and the score transformed in to a scale from 0 to 100, with a higher score indicating better usability (Intervention arm only).

7. SIGNIFICANCE AND TRANSFORMING POTENTIAL

This study will be the first in Ethiopia, and of the three in sub-Saharan Africa, to evaluate the effectiveness of pillbox-enabled SAT as a multicenter randomized controlled trial. The study will provide evidence of whether the pillbox-enabled SAT is non-inferior to the standard DOT for medication adherence and treatment outcomes. The study will assess whether digital adherence intervention is cost-effective, usable, and acceptable. The study targets TB patients living in Ethiopia - a high TB burden LMIC in Sub-Saharan Africa where alternative TB treatment strategies to in-person DOT do not exist.

If effective, this approach could substantially improve treatment adherence, increase sputum conversion, reduce patient-side costs due to daily DOT, and improve quality of life. It could also have a strong public health impact by reducing transmission of the disease to healthcare providers and the community at large as the approach can reduce patients' daily travels to healthcare facilities for in-person DOT. This approach will provide TB patients freedom and ownership of their treatment, which ultimately reduces mortality and morbidity thereby contributing significantly to the End TB Strategy. The findings will enable patients, healthcare providers, and policymakers to make informed decisions about the value of the intervention.

The study intends to use several clinical, biomedical, behavioral, and economic measurement tools to learn extensively about the desired outcomes of interest. The assessment of adherence using electronic, self-report, and biological specimens will provide credible and reliable justification of results. The diagnostic tools we plan to use are WHO-approved, and the questionnaires are valid and psychometrically sound for use in a local context. The study will collect original data from both patients and their TB care providers in local facilities, providing substantial evidence on the usability and acceptability of the intervention in real-world settings.

If the intervention arm is found non-inferior to DOT, the investigators will present the findings to the Ethiopian Federal Ministry of Health and work with the Bureau to scale up the intervention to larger

healthcare facilities in Ethiopia. The investigators will also work with the World Health Organization and donor agencies to see and discuss the scalability of the intervention to other high TB burden countries in Sub-Saharan Africa.

8. METHODS

8.1. Materials

The equipment for this study is an electronic pillbox, evriMed500 digital medication monitoring and reminder device manufactured by Wisepill Technologies, South Africa (Figure 5) [92]. It was developed with funding from the Bill & Melinda Gates Foundation and is currently in use in clinical trials and in clinical practice. The evriMED medication adherence device is an affordable and TB-appropriate digital medication adherence tool that has been tested extensively and is being used in India and China for TB patients (92). It costs less than 10 USD per patient based on conservative reuse assumptions, and it could easily be integrated into existing national health data systems [93]. The device is TB-appropriate technology to permit customization of the container for Drug-susceptible-TB, MDR-TB and TB-HIV patients.

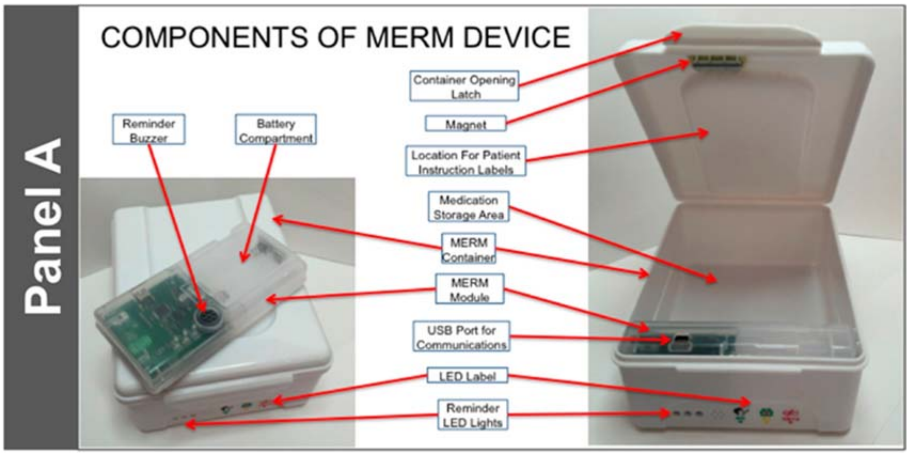

Figure 5: Electronic pillbox for adherence monitoring: components of the MERM module and its location in the container in which the medication blister cards are stored (91)

The evriMED500 dispenser consists of two hardware components, namely the electronic module and the medication container. This modular design allows the electronic module to be reused and the container to be replaced if needed. The electronic module slots into the container so that the indicator LEDs are visible through the front of the container. A USB port can be accessed by opening

the container. The electronic module has a USB port for downloading data and for configuration of the unit.

The container has three Indicator Lights/LEDs (green, yellow, and red). The green LED will flash once when the container is opened and again once when the container is closed; will quickly flash three times when the container is opened and closed quickly; will flash in sequence during the (daily) Medication Alarm; and will be on solid, while connected via USB to the computer. The Yellow LED is to refill medication and will flash with the Green LED at the time of the medication alarm. If the Medication Alarm is not enabled, only the yellow LED will flash. It will be on solid when the container is opened. The Red LED flash is for the battery, and it will flash with the Green LED at the time of the Medication Alarm. It will be on solid when the container is opened.

The device has a medication intake event in an electronic record that is created when a patient opens the device to take his or her medication. It consists of a date-and-time stamp. The dispenser provides an electronic heartbeat to a central management system whenever medication is taken, and this also indicators that the device was functioning during that period.

The alarm period is 30 minutes in length and consists of three-alarm cycles. The alarm cycles are 10 minutes each and made up of two parts. These alarms apply regardless of the type of reminder selected. The first five minutes of the alarm cycle is the active alarm. During the active alarm, the buzzer will sound, and the green LED will flash in the following sequence: short, short, long. The yellow LED will flash if the “Refill Alarm” is configured and the date is less than or equal to the current date. The second five minutes of the alarm cycle is the passive alarm. During the passive alarm, the buzzer will not sound, and the green LED will not flash. The yellow LED will continue to flash if the “Refill Alarm” is configured and the date less than or equal to today. Opening the container will cancel the active alarm if it is opened within four hours before the scheduled alarm or if it is opened during the alarm period.

The evriMED500 makes use of two AA batteries which should last for more than 12 months. Regarding storage capacity, it can store more than 12000 records/events. The evriMED PC Application is configured by the evriMED PC Application and it allows a direct connection to the evriMED device via a USB cable. Specification of the evriMED500 is summarized in table 2.

Table 2: Summary of evriMED500 specification

Item	Specification
Data Storage	12000 records
Battery Life (Alkaline)	12+ months
Power requirements	Two AA batteries
Weight: Module + Standard Battery and Standard Container	280g
Dimension: Standard Container	166.9 mm x 129 mm x 71.4 mm
Dimension: Module	119.8 mm x 60.6 mm x 19.5 mm
Material	Polypropylene Copolymer
Temperature range	The Operating range is 0 to +50°C The storage temperature is 0 to +70°C
Relative Humidity	20% to 65% non-condensing
Shock Resistance	Withstands a one-meter drop onto a solid surface
Protection from Liquids and Dust	Dust and splash resistant
Vibration	10 ~ 55Hz and amplitude 0.35mm
Safety	IEC 60950-1:2013

8.2. Trial design

The study will be a prospective, multicenter, randomized, controlled, non-inferiority, effectiveness-implementation hybrid type 2, two-arm trial. The study will not dictate diagnosis or treatment for TB; thus, it will not introduce or use new medications. Systematic and desk review methods will guide the study aimed at exploring the digital health ecosystem in Ethiopia.

8.3. Setting

Ethiopia is structurally divided into 11 autonomous administrative divisions. Addis Ababa, the capital, is among these administrative divisions and is the largest city of Ethiopia with the status of both a city

and state. Addis Ababa is considered by some to be the capital of Africa, as it is a seat for the African Union headquarters and other international and regional organizations including the Africa Centers for Disease Control and Prevention. Administratively, Addis Ababa is divided into 10 sub-cities with distinct locations. As the city is crowded with population and housing, there is a high risk of TB transmission. The national health and health-related indicator [94] reports that in 2016, 2,290 bacteriologically confirmed TB cases were identified in the city, of which 1,811 (79.1%) completed their treatment and 1,526 (66.6%) were cured. For these reasons, Addis Ababa was chosen as the study setting.

8.4. Site selection

In Addis Ababa, a total of 94 public health centers provide TB care and treatment services under the DOT program. This study stratifies the 94 health centers on the bases of the 10 sub-cities where they are located. From each stratified group, one health center with the largest TB client load will be selected, with a total of 10 health centers to be included. Health management information system (HMIS) quarterly data (April 01 - June 30, 2019) taken from the Addis Ababa Regional Health Bureau will be used for the purpose. This will give a representative sample of study sites and participants. A TB clinic in each center will serve as the study site and primary location for patient contact for that center. Table 3 lists outs the 10 public health centers.

Table 3: Study sites

S. No.	Name of Health Center	Sub-city	# PTB cases bacteriologically confirmed or referred from other sites for initiation of TB treatment, April 01 - June 30, 2019
1.	Addis Raey Health Center	Addisketema	41
2.	Akaki Health Center	Akaki kality	47
3.	Kebena Health Center	Arada	14
4.	Goro Health Center	Bole	31
5.	Adisu Gebeya Health Center	Gulele	22
6.	Kazanchis Health Center	Kirkos	19
7.	Alem Bank Health Center	Kolfe	38
8.	Teklehaymanot Health Center	Lideta	22
9.	Woreda 02 Health Center	Nifasilk lafto	53
10.	Woreda 13 Health Center	Yeka	35

The sub-cities selected should be willing and capable of participating in the study. If any health facility fails to do so, the study will consider the next facility with a large TB patient load. TB clinics in each of the study sites will be the primary sites for patient contact.

The study will be held as a collaborative undertaking between Addis Ababa University (Ethiopia) and Emory University (USA), and investigators from both institutions will take part in the study.

8.5. Study participants

The study participants are TB patients and their healthcare providers, while the primary participants are TB patients. Objectives of the study related to medication adherence and treatment outcomes will rely solely on data collected from patients, while objectives related to usability, cost-effectiveness, acceptability, and behavioral and socio-cultural factors will rely on data collected from both patients and providers.

8.5.1. Study participants (TB patients)

8.5.1.1. Study population

The efficacy studies target TB patients. The source population will be all new patients with TB symptoms who come to a study site and undergo bacteriological screening during the study period or TB patients bacteriologically confirmed elsewhere and referred to a study site for TB treatment.

8.5.1.2. Inclusion criteria

Inclusion criteria will include:

- Patients with new, or previously treated, bacteriologically-confirmed drug-sensitive pulmonary TB
- Eligible to start the standard 6-month first-line anti-TB medication
- Outpatient status at the time of screening and enrollment
- Men or women age >=18 years
- Able and willing to provide informed consent

8.5.1.3. Exclusion criteria

The exclusion criteria will include:

- Patients with known DR-TB
- Any condition that causes cognitive impairment such as severe acute illness or injury, developmental retardation, or severe psychiatric illness and thus precludes informed consent or safely participating in the study procedures
- Inpatient status at the time of screening and enrollment
- Expected to move away from the study site or become incarcerated before the final study follow up at month two
- Concurrent extra-pulmonary TB
- Contraindicated medications
- Active liver disease that requires a TB regimen other than HREZ - isoniazid, rifampicin, pyrazinamide, and ethambutol.

8.5.1.4. Justification for inclusion and exclusion

DR-TB patients take TB medications over a long time and as an inpatient, thus are unable to experience SAT. Similarly, inpatients, as they are admitted and assigned a bed to receive treatment and care, are unable to effectively experience the SAT.

8.5.1.5. Sampling and sample size

The sample size is calculated considering a 1-sided type I error of 2.5%, a power of 80%, 10% attrition rate, delta of 20%, non-inferiority margin, and a continuous outcome of percentage adherence over the two-month intensive phase, with a standard deviation of 36% and 79% of average adherence [31], assuming null hypothesis for both arms. The results yield a sample size of 57 in each arm for a total of 114 participants. We will do a $\log_{10}$ transformation on the data, where a difference can be equivalently transformed into a ratio using a power (10^a). The non-inferiority will be calculated as the log in the control minus the log in the intervention, which is equivalent to the log of the control divided by intervention.

8.5.2. Provider participants

8.5.2.1. Study population

Here, the source population will be healthcare providers who give TB treatment in public health care facilities. The study population is all adults in the study sites who are currently providing TB treatment under DOT.

8.5.2.2. Inclusion criteria

Inclusion criteria will include:

- A healthcare provider with academic qualification as a medical doctor, health officer (BSc), or nurse (BSc or Diploma);
- At least three months of experience providing DOT services in the facility;
- Able and willing to provide informed consent

8.5.2.3. Exclusion criteria

The exclusion criteria are:

- Not trained or is not familiar with DOT,
- Expected to move away from the facility before completion of enrolment of study patients

8.5.2.4. Justification for inclusion and exclusion

Health care providers who are not trained on DOT or do not have experience of the service would not be able to share their experience on DOT and handle patients per the need. A healthcare provider who is already known to leave the facility before completion of the study would interrupt the data collection process and affect the quality and outcome of the study.

8.5.2.5. Sampling and sample size

The study intends to involve 10 health facilities. Thus, a total of 10 healthcare providers, one from each, who are providing DOT in the data collection period will be identified purposively and enrolled. A list of reserves, one from each facility, will be kept to engage in case of any provider missing.

8.6. Interventions

Participants in the intervention arm will receive a 15-days TB medication supply (HRZE fixed-dose combination therapy of 15 doses) in an electronic pillbox device (evriMed500 digital medication monitoring and reminder device manufactured by Wisepill Technologies, South Africa) to self-administer. Providers will collect baseline data, including demographic, socioeconomic, behavioral, and social factors using the study's baseline questionnaire. Based on the baseline data, participants will be clustered into four behavioral determinants (use cigarettes, alcohol, Khat - a psychostimulant plant, and cocaine/marijuana) and three social determinants (homeless, unemployed, and illiterate) as

appropriate for the purpose of analysis. Participants in this arm will return every 15 days, where the provider will count any remaining tablets in the pillbox, download the pill-taking data from the Wisepill device, evaluate the functionality of the device and troubleshoot as needed, and perform the urine isoniazid test. The level of adherence in their intensive phase of treatment will be calculated using the medication possession ratio (MPR) [91]. Any participant who misses more than five tablets in any 15-day refills will be reassigned to daily DOT throughout the remaining days of the intensive phase. Participants in the intervention arm can consult the healthcare provider in cases of medical illness or any adverse events outside of a scheduled visit before the next appointment. The phone number of the healthcare provider following their TB condition will be written at the backside of their appointment cards. The phone call strategy aims to maintain the DOT advantage for the intervention arm.

Participants in the control arm will get their treatment as per the standard practice of DOT, where participants will visit the healthcare facility each business day throughout the two months intensive phase to swallow their daily dose of HRZE with direct observation by the healthcare provider. Additionally, they will be given pills for the weekend to take them at home. The provider will collect baseline data, conduct a urine isoniazid test every 15-days, and cluster participants into behavioral and social determinants as applicable for participants in the intervention arm. For this arm, the management of participants who interrupt treatment will follow the national TB treatment guidelines.

Both arms will be followed up throughout the intensive phase which lasts for two months. The continuation phase (4 months) will follow the standard DOT practice for both arms. Both arms will have a TB care and treatment service free of charge, and the pillbox will be given to each participant in the intervention arm free of charge. For both arms, participants will receive treatment according to Ethiopian national TB treatment guidelines.

8.7. Study procedure

The main data collection tools will include

- a baseline patient information questionnaire (demographic, socioeconomic, behavioral, social, and clinical information),
- medication adherence measurement tools (MERM vs. DOT daily treatment adherence monitoring tool and urine colorimetric isoniazid test - IsoScreenTM test, GFC Diagnostics Ltd, Bicester, England, and adherence self-report questionnaire),

- clinical measurement tools (pre-post treatment sputum Xpert MTB/RIF assay or microscopy and adverse treatment outcome monitoring tool),
- cost/economic tools (health-related quality of life [HRQoL], catastrophic cost, post-diagnostic cost from an individual patient's perspective, predictors of HRQoL and catastrophic costs,
- Usability and treatment satisfaction tools (treatment satisfaction questionnaire for medication version 1.4 (TSQM v1.4) tool and an 18-item treatment satisfaction questionnaire

The study investigators will identify healthcare providers in the TB clinics and provide training on the study procedures, how to operate the evriMED500 device, and perform a urine isoniazid test. The investigators will perform a 1:1 randomization of participants before the start of the study using computer-generated random numbers. Providers will enroll participants in the two arms sequentially as they arrive in the clinic and the investigators will routinely monitor the process. Providers will provide instruction using the Amharic version of the study's participant information sheet. Providers will recruit participants and obtain written informed consent in the local language, which is Amharic. The consent form will also function as an acknowledgment of personal responsibility keeping the device properly and returning upon completion of the study.

For both arms, the providers will collect baseline data, including demographic, socioeconomic, behavioral, and social determinants social using the study' baseline questionnaire. For participants in the intervention arm, the providers will orient participants in the intervention arm as they enroll about how to use the evriMED500 device. The orientation time will depend on the efficiency of the participants to fully acquire and demonstrate the necessary skills. Providers will then dispense a 15-days TB medication supply (HRZE fixed-dose combination therapy of 15 doses) to participants within the evriMed500 device for self-administration. Providers will collect sputum specimens from participants; write their cell phone numbers at the backside of participants' appointment cards to communicate in cases of medical illness or any adverse events before the next appointment, and inform them to return every 15 days. When participants returned, the providers will count any remaining tablets in the pillbox, download the pill-taking data from the evriMED device, check functionally of the device, and conduct a urine isoniazid test. The providers will fill out the study's adherence follow-up form to capture information if the drugs are taken every day, and if not, the reasons for non-adherence. The providers will then evaluate the level of adherence based on the preset criteria, and refill a 15-day TB medication supply in the same pillbox as appropriate. The providers will collect sputum specimens from participants at the end of the intensive phase.

For the control arm, providers will handle participants according to the standard DOT procedure, where participants will visit the healthcare facility each business day in the intensive phase to swallow their daily dose of HRZE with direct observation by the provider. The providers will fill out a similar adherence follow-up form for the control arm. Additionally, if a participant in the control arm requests medications for self-administration, the provider will collect information about the date requested and for how many days requested on the follow-up form.

For the control arm, in addition to monitoring medication adherence, a separate secondary analysis will be conducted for any doses that are self-administered after approval from their provider to determine if this approach has an impact on other outcome measures. This will be to determine the real-world practice of in-person DOT where some doses might be self-administered when the provider approved this procedure for extenuating circumstances.

The participants will have IsoScreenTM urine isoniazid test every 15 days, which is a colorimetric assay, whereby the pyridine ring structure of isoniazid and its metabolites is broken by the biochemical reaction leaving it vulnerable to attachment by the condensing agent, barbituric acid.

At the end of the intensive phase, trained research experts will complete several data instruments for all participants. The first is a self-report of medication adherence that the experts will administer. The second is a case report form for which data will be extracted from TB registration logs and participants' charts, focusing on overall treatment outcomes and side effects. For the third instrument, the experts will administer the EuroQol's EQ-5D-5L HRQoL questionnaire to all participants. The experts will also assess patients' costs using the Tool to Estimate Patients' Costs. Then the experts will administer the TSQM version 1.4 tool to determine treatment satisfaction. Finally, the experts will administer the 18-item tool to determine user performance of the MERM device for intervention participants. Following the collection of data from participants, the study investigators will collect qualitative and quantitative data from the providers to learn more about the usability and acceptability of the intervention from providers and the healthcare system perspectives. The investigators will follow-up and capture ongoing data on the status of each pillbox, thus from the distribution of the pillboxes to end of data collection.

For the laboratory research involving biological specimens (urine and sputum), the study will use WHO-approved diagnostic tools. The IsoScreen test is a semi-quantitative urine test that provides a reliable and immediate indication of adherence to isoniazid-containing treatment regimens for

patients with TB. It uses the reagents of the Arkansas Method: barbituric acid (20 mg), potassium cyanide (10 mg) and chloramine-T (10 mg), in an enclosed plastic testing device for safe and rapid testing in clinics and patients' homes. A urine sample is added to the plastic testing device. If the sample develops a blue color, it is positive for isoniazid metabolites and is therefore from a patient who is compliant with the treatment. A blue/purple color indicates that the drug was taken within the last 24 hours. A green color also indicates isoniazid metabolites, but the drug was probably taken about 48 hours ago. If the sample remains yellow, then the patient is not adhering to their daily treatment. The providers will perform this test within the study facilities for both arms every 15 days, thus four times per participant. Sputum specimens collected before and after the intensive phase. Under the routine practice, the sputum samples will be tested for Acid-fast bacilli (AFB) microscopy to check for sputum smear conversion following the intensive phase. Initiation, termination or completion of treatment will rely only on the standard procedures available at the study sites, which could be acid-fast bacilli or Xpert MTB/RIF assay. Thus, the outcomes of the study's laboratory results will not dictate the standard diagnostic or treatment procedures.

8.8. Definition of terms

8.8.1. Medication adherence

- The extent to which patients take their medications as prescribed with respect to dosage and dosage intervals throughout the treatment period (70, 100)

8.8.2. Effectiveness

- The ability of a digital health intervention to achieve the intended results (70)

8.8.3. Cost-effectiveness

- Comparison of two alternatives where consequences of the intervention are measured in natural units (70)

8.8.4. Usability

- the capability in human functional terms to be used easily and effectively by the specified range of users, given specified training and user support, to fulfill the specified range of tasks, within the specified range of environmental scenarios (70).

8.8.5. TB patient

- An individual diagnosed with active TB disease (pulmonary or extrapulmonary) (101).

8.8.6. TB patient cost

- Survey of costs faced by TB-affected patients and their households (102).

8.8.7. New cases

- A newly registered episode of TB in a patient who has never been treated for TB or has taken anti-TB medicines for less than 1 month (103).

8.8.8. Bacteriologically confirmed TB case

- A patient from who has at least one positive result either by smear microscopy, culture or Xpert MTB/RIF assay (46).

8.8.9. Loss to follow-up

- Patients have previously been treated for TB and were declared lost to follow-up at the end of their most recent course of treatment and are now diagnosed with TB (46).

8.8.10. Treatment completed

- A TB patient who completed treatment without evidence of failure BUT with no record to show that sputum smear or culture results in the last month of treatment and on at least one previous occasion were negative, either because tests were not done or because results are unavailable.

8.8.11. Healthcare facility

- Any establishment of facility (public or private) that is engaged in direct care of patients on site (101).

8.9. Data management

The study will use a password-protected offline Research electronic data capture (REDCap) database, Vanderbilt University, United States [105] to enter data and store entered data in an encrypted drive.

The Principal Investigator will keep the source data in a locked cabinet at the study's central office. The assigned study staff will check a random sample of 10% of all data entry forms for entry errors.

9. STATISTICAL ANALYSIS

Descriptive summary measures will be used to report participant characteristics. Chi-square tests will be used to evaluate potential associations among categorical variables. To compare the level of adherence between study arms and among variables, independent t-tests will be done on log-transformed adherence percentage of the expected 60 days. Effects of the arms and other adherence variables will be estimated using a geometric mean (GM) with geometric standard deviation (GSD) and mean ratios (MR) with 95% confidence intervals (CI). Log binomial regression will be conducted to identify risk factors of at least one negative isoniazid urine test and self-reported adherence of participants. Effects will be measured using adjusted relative risk (ARR) with 95%CI. A general linear model will be done on log-transformed adherence percentage to identify the effects of variables on participants' level of adherence. Effects will be measured using an adjusted mean ratio (AMR) with 95% CI.

Descriptive statistics including frequency and percentage will be used to describe the health state of the study participants. Multiple bar charts with cross-tabulation will be used to illustrate distributions of health states by study arms. Chi-square and Fisher's exact tests will be employed to compare the five EQ-5D-5L health domains by study arms. Kolmogorov–Smirnov, Shapiro–Wilk tests, and median values with interquartile range (IQR) will be used to summarize EQ-5D-5L index value/utility. Nonparametric Mann–Whitney U test will be employed to compare the difference in EQ-5D-5L index value/utility among study arms. A log-binomial model will be used to identify risk factors for lower HRQoL, which is having at least one health problem.

The overall TB treatment cost will be estimated by considering costs related to anti-TB drug pick-up, guardian costs, and coping costs over the two-month intensive phase. The proportion of study participants who faced catastrophic costs at a cut-off point of 20% will be estimated. A cross-tabulation will be employed to evaluate the distribution of catastrophic cost over the study arms and chi-square test will be used to test the association between catastrophic cost and study arms.

To compare the TSQM scores between study arms, independent t-tests will be done on log-transformed scores. Effects of the arms will be estimated using a geometric mean (GM) with geometric standard deviation (GSD) and mean ratios (MR) with 95% confidence intervals (CI). A

general linear regression will be done on log-transformed TSQM scores to identify the effects of variables on participants' level of satisfaction. Effects will be measured using an adjusted mean ratio (AMR) with 95% CI. In all analyses, a 5% significance threshold will be used to determine statistical significance.

10.QUALITY ASSURANCE

The investigators will generate, document, and report the trial in compliance with the protocol, GCP guideline, and applicable national and international (US NIH) regulatory requirements. External monitors may do technical audits, before, during, and after completion of the trial. This will include reviewing the research protocol, operations manual, standard operating procedures, and training materials before initiation of the trial. The site investigators will make study documents and pertinent records readily available for inspection by the local IRB and site monitors. The study sponsor may conduct monitoring or auditing of study activities to ensure the scientific integrity of the study and to ensure the rights and protection of study participants. Monitoring and auditing activities may be conducted by the sponsor (internal), authorized representatives of the sponsor (external) or both. Monitoring or auditing may be performed by means of on-site visits to the Investigators' facilities or through other communications such as telephone calls or written correspondence. The visits will be scheduled at mutually agreeable times, and the frequency of visits will be at the discretion of the sponsor. During the visit, any study-related materials may be reviewed and the Investigators along with study healthcare providers will be available for discussion of finding. The study may also be subject to inspection by regulatory authorities (national or foreign) as well as the IRBs to review compliance and regulatory requirements. Given that the study is neither dictating the need for or treatment of TB diagnosed by the screening, serious adverse events (SAEs) will not be directly attributable to the study.

11.STUDY ASSESSMENT AND DISCONTINUATION

11.1. Participant discontinuation

- There will be premature study discontinuation if there is:
- Refusal of study participants to participate in all components of the study;
- A request by the participants to withdraw;
- A request from the healthcare providers if s/he thinks the study is no longer in the best interest of the participants; or

- At the discretion of the IRB/Ethics Committee, regulatory bodies, sponsor or consensus of the investigators.

11.2. Unexpected or adverse events

Given that the study is neither dictating the need for treatment of TB or specific regimen, serious adverse events will not be directly attributable to the study. The study will not dictate diagnosis or treatment algorithms for TB, and all diagnoses testing assays and treatment regimens will follow the Ethiopian national guidelines; thus, it will not introduce or prescribe new drugs. The major risks of this screening program are related only to pill-taking mechanisms. Both adverse events and serious adverse events will be reported to the EFDA in line with the stipulated timeline irrespective of relatedness to the study procedure. Adverse reactions to TB medications will not be considered the outcomes of the study. The investigators will capture these events only to the extent they are available in the study health facilities' records and registries. The investigators will conduct chart abstractions to review adverse events that are related to TB treatment, as recorded from the TB clinic registries. For patients who are co-infected with HIV, the chart review will also include the type of HIV regimens, viral load, CD4 counts, and other co-infections diagnosed and treated.

Regarding TB diagnosis, at the initial stage or following the intensive phase, there could be discrepancies between the health facility results (Smear microscopy or Xpert MTB/RIF) and the study result (MGIT liquid culture). In this case, the investigators will communicate the results to the healthcare providers for their review and decision.

11.3. Interim monitoring and analysis

The investigators will conduct interim monitoring and submit an analysis report to the independent Data and Safety Monitoring Board (DSMB). Then, the report will be sent together with DSMB's recommendation to the IRBs. The DSMB will periodically review and evaluate the study's collected data to follow up participant safety, the accuracy of study procedures, and the study progress in order to provide recommendations on the continuation, modification, or termination of the study. The DSMB will consist of an expert in the clinical aspects of TB, an expert biostatistician, and an investigator with expertise in current clinical trials conduct and methodology. External monitors may conduct technical audits, before, during, and after completion of the trial. This will include reviewing the research protocol, operations manual, standard operating procedures, and training materials

before initiation of the trial. The site investigators will make study documents and pertinent records readily available for inspection by the local IRB and site monitors.

12.ETHICAL CONSIDERATIONS

12.1. Statement of compliance

The trial will be carried out in accordance with the International Conference on Harmonization Good Clinical Practice (ICH GCP) and the recently adopted (2018) good clinical trial guideline and requirements of the Ethiopian Food and Drug Administration (EFDA) (30). The investigators have completed Human Subjects Protection and ICH GCP Training. Clinical trial site staff who are responsible for the conduct, management, or oversight of the trial will complete Human Subjects Protection and ICH GCP training. The protocol, informed consent forms, recruitment materials, and all participant materials will be submitted to the Institutional Review Board (IRB) for review and approval. Approval of both the protocol and the consent form will be obtained before any participant is enrolled. Any amendment to the protocol will be reviewed and approval by the IRB before the changes are implemented to the study. In addition, all changes to the consent form will be IRB-approved; a determination will be made regarding whether a new consent needs to be obtained from participants who provided consent, using a previously approved consent form.

12.2. Ethical review

The study does not involve new investigational product and no exemption will be sought. The pillbox device has been in use for clinical purposes other than TB. There will be no change in the treatment of TB from the national guidelines, neither the type of regimen nor the dose, but only the management of treatment.

The proposal and any subsequent modifications will be reviewed and approved by:

i) Scientific and Ethics Review Committee of the Center for Innovative Drug Development and Therapeutic Trials for Africa, College of Health Sciences, Addis Ababa University;
ii) Institutional Review Board of the College of Health Sciences, Addis Ababa University;
iii) Ethiopian Food and Drug Administration Authority (EFDA);
iv) Ethiopian National Research Ethics Review Committee (NRERC); and
v) The Institutional Review Board of Addis Ababa City Administration Health Bureau

All study sites will have their permission and written concurrence for data collection

12.3. Informed consent

Study participants, both patients and providers, will be given an information sheet, which includes the purpose of the study, the procedures to be followed, privacy and confidentiality and the risks and benefits of participation. The information sheet will be read to those who cannot read or write in the presence of a witness. All questions about the study will be answered to the satisfaction of the participant. If they agree to be part of the study, they will sign the informed consent. If the participant is illiterate, they will thumbprint the consent form, including the signature of the witness. A copy of the information sheet and the signed consent form will be given to each participant. The interview questionnaire will be conducted and filled in private. Neither the investigators nor the study provider data collectors will coerce or unduly influence the potential patient to participate or to continue to participate in the study. Participants will have the right to withdraw without any reason for termination or cross-over, and that care will not be affected in any way by declining or participating in the study.

12.4. Confidentiality

All filled questionnaires, evaluation forms, reports, and other records that leave the study sites will be identified by coded numbers only to maintain subject confidentiality. All records will be kept locked at the principal investigator’s study office in Addis Ababa University, CDT-Africa. All computer entry and networking programs will be locked with a password. Participant’s information will not be released without written permission of the subject, except as necessary for monitoring by the study team. Only the investigators will have access to the crude data.

12.5. Payment for participation

There will be no payment for participation, either in cash or in-kind, to patients in the intervention or DOT arm, or healthcare providers, as this would influence outcomes of the study. However, if the participants are coming just for the purpose of the study, they will be reimbursed for transport.

13.REFERENCES

1. Jiang WX, Long Q, Lucas H, Dong D, Chen JY, Xiang L, et al. Impact of an innovative financing and payment model on tuberculosis patients' financial burden: is tuberculosis care more affordable for the poor? Infect Dis Poverty. 2019;8(1):21.
2. Ukwaja KN. Social protection interventions could improve tuberculosis treatment outcomes. Lancet Glob Health. 2019;7(2):e167-e168.
3. Richterman A, Steer-Massaro J, Jarolimova J, Luong Nguyen LB, Werdenberg J, Ivers LC. Cash interventions to improve clinical outcomes for pulmonary tuberculosis: systematic review and meta-analysis. Bull World Health Organ. 2018;96(7):471-483.
4. Shete PB, Reid M, Goosby E. Message to world leaders: we cannot end tuberculosis without addressing the social and economic burden of the disease. The Lancet Global Health. 2018;6(12):e1272-e3.
5. Dara M, Zachariah R. Hunger and tuberculosis: Two sides of the same coin. International Journal of Tuberculosis and Lung Disease. 2018;22(6):592.
6. Sakamoto H, Lee S, Ishizuka A, Hinoshita E, Hori H, Ishibashi N, et al. Challenges and opportunities for eliminating tuberculosis - leveraging political momentum of the UN high-level meeting on tuberculosis. BMC Public Health. 2019;19(1):76.
7. Ki-Moon B. Building a tuberculosis-free world on a foundation of universal health coverage. Lancet. 2019;393(10178):1268-1270.
8. Agins BD, Ikeda DJ, Reid MJA, Goosby E, Pai M, Cattamanchi A. Improving the cascade of global tuberculosis care: moving from the "what" to the "how" of quality improvement. Lancet Infect Dis. 2019 Aug 22. approved: S1473-3099(19)30420-7[Epub ahead of print].
9. Reid MJA, Arinaminpathy N, Bloom A, Bloom BR, Boehme C, Chaisson R, et al. Building a tuberculosis-free world: The Lancet Commission on tuberculosis. Lancet. 2019;393(10178):1331-1384.
10. Cohen KA, Manson AL, Abeel T, Desjardins CA, Chapman SB, Hoffner S, et al. Extensive global movement of multidrug-resistantM. tuberculosis strains revealed by whole-genome analysis. Thorax. 2019;74(9):882-889.

11. Chihota et al, 2018. Geospatial distribution of Mycobacterium tuberculosis genotypes in Africa. PLoS One. 13(8):e0200632.
12. Knight GM, McQuaid CF, Dodd PJ, Houben RMGJ. Global burden of latent multidrug-resistant tuberculosis: trends and estimates based on mathematical modelling. Lancet Infect Dis. 2019;19(8):903-912.
13. Walker TM, Merker M, Knoblauch AM, Helbling P, Schoch OD, van der Werf MJ, et al. A cluster of multidrug-resistant Mycobacterium tuberculosis among patients arriving in Europe from the Horn of Africa: a molecular epidemiological study. Lancet Infect Dis. 2018;18(4):431-40.
14. Wild V1, Jaff D2, Shah NS3, Frick M. Tuberculosis, human rights and ethics considerations along the route of a highly vulnerable migrant from sub-Saharan Africa to Europe. Int J Tuberc Lung Dis. 2017;21(10):1075-85
15. Armstrong LR, Winston CA, Stewart B, Tsang CA, Langer AJ, Navin TR. Changes in tuberculosis epidemiology, United States, 1993-2017. Int J Tuberc Lung Dis. 2019;23(7):797-804.
16. Eshetie S, Gizachew M, Alebel A, van Soolingen D. Tuberculosis treatment outcomes in Ethiopia from 2003 to 2016, and impact of HIV co-infection and prior drug exposure: A systematic review and meta-analysis. PLoS One. 2018;13(3):e0194675.
17. Matteelli A, Rendon A, Tiberi S, Al-Abri S, Voniatis C, Carvalho ACC, et al. Tuberculosis elimination: where are we now? Eur Respir Rev. 2018;27(148).
18. Collins D, Hafidz F, Mustikawati D. The economic burden of tuberculosis in Indonesia. Int J Tuberc Lung Dis 2017; 21;1041-8.
19. Tanimura T, Jaramillo E, Weil D, Raviglione M, Lonnroth K. Financial burden for tuberculosis patients in low- and middle income countries: a systematic review. Eur Respir J 2014;43:1763-75.
20. van den Hof S, Collins D, Hafidz F, Beyene D, Tursynbayeva A, Tiemersma E. The socioeconomic impact of multidrug resistant tuberculosis on patients: results from Ethiopia, Indonesia and Kazakhstan. BMC Infect Dis 2016;16:470.
21. Harries AD, Schwoebel V, Monedero-Recuero I, Aung TK, Chadha S, Chiang CY, et al. Challenges and opportunities to prevent tuberculosis in people living with HIV in low-income countries. Int J Tuberc Lung Dis. 2019;23(2):241-51.
22. Diallo A, Dahourou DL, Dah TTE, Tassembedo S, Sawadogo R, Meda N. Factors associated with tuberculosis treatment failure in the Central East Health region of Burkina Faso. Pan Afr Med J. 2018;30:293.

23. Ruru Y, Matasik M, Oktavian A, Senyorita R, Mirino Y, Tarigan LH, et al. Factors associated with non-adherence during tuberculosis treatment among patients treated with DOTS strategy in Jayapura, Papua Province, Indonesia. Glob Health Action. 2018;11(1):1510592.
24. Gebreweld FH, Kifle MM, Gebremicheal FE, Simel LL, Gezae MM, Ghebreyesus SS, et al. Factors influencing adherence to tuberculosis treatment in Asmara, Eritrea: a qualitative study. J Health Popul Nutr. 2018;37(1):1.
25. Kibuule D, Verbeeck RK, Nunurai , Mavhunga F, Ene E, et al. Predictors of tuberculosis treatment success under the DOTS program in Namibia. Expert Rev Respir Med. 2018;12(11):979-87.
26. Sharma SK, Jha S. Directly observed treatment is not the only solution for poor adherence and low cure of tuberculosis. Evid Based Med. 2015;20(5):180.
27. World Health Organization (WHO). Adherence to long-term therapies: evidence for action. WHO; Geneva, Switzerland, 2003
28. Pérez-Jover V, Sala-González M, Guilabert M, Mira JJ. Mobile Apps for Increasing Treatment Adherence: Systematic Review. J Med Internet Res. 2019;21(6):e12505.
29. World Health Organization (WHO). WHO TB report 2018. WHO: Geneva, Switzerland; 2018.
30. Collins D, Beyene D, Tedla Y, Mesfin H, Diro E. Can patients afford the cost of treatment for multidrug-resistant tuberculosis in Ethiopia? Int J Tuberc Lung Dis. 2018;22(8):905-11.
31. Asres A, Jerene D, Deressa W. Pre- and post-diagnosis costs of tuberculosis to patients on Directly Observed Treatment Short course in districts of southwestern Ethiopia: a longitudinal study. J Health Popul Nutr. 2018 May;37(1):15.
32. Woimo TT, Yimer WK, Bati T, Gesesew HA. The prevalence and factors associated for anti-tuberculosis treatment non-adherence among pulmonary tuberculosis patients in public health care facilities in South Ethiopia: a cross-sectional study. BMC Public Health. 2017;17(1):269.
33. Getahun B, Wubie M, Dejenu G, Manyazewal T. Tuberculosis care strategies and their economic consequences for patients: the missing link to end tuberculosis. Infect Dis Poverty. 2016;5(1):93.
34. Mesfin EA, Beyene D, Tesfaye A, Admasu A, Addise D, Amare M, et al. Drug-resistance patterns of Mycobacterium tuberculosis strains and associated risk factors among multi drug-resistant tuberculosis suspected patients from Ethiopia. PLoS One. 2018;13(6):e0197737.

35. Fiseha D, Demissie M. Assessment of Directly Observed Therapy (DOT) following tuberculosis regimen change in Addis Ababa, Ethiopia: a qualitative study. BMC Infect Dis. 2015;15:405.
36. Genet C, Melese A, Worede A. Effectiveness of directly observed treatment short course (DOTS) on treatment of tuberculosis patients in public health facilities of Debre Tabor Town, Ethiopia: retrospective study. BMC Res Notes. 2019;12(1):396.
37. Getahun B, Nkosi ZZ. Is directly observed tuberculosis treatment strategy patient-centered? A mixed method study in Addis Ababa, Ethiopia. PLoS One. 2017;12(8):e0181205.
38. Sagbakken M, Frich JC, Bjune GA, Porter JD. Ethical aspects of directly observed treatment for tuberculosis: a cross-cultural comparison. BMC Med Ethics. 2013;14:25.
39. Getahun B, Nkosi ZZ. Satisfaction of patients with directly observed treatment strategy in Addis Ababa, Ethiopia: A mixed-methods study. PLoS One. 2017;12(2):e0171209.
40. Adenager GS, Alemseged F, Asefa H, Gebremedhin AT. Factors Associated with Treatment Delay among Pulmonary Tuberculosis Patients in Public and Private Health Facilities in Addis Ababa, Ethiopia. Tuberc Res Treat. 2017;2017:5120841.
41. Tadesse S. Stigma against Tuberculosis Patients in Addis Ababa, Ethiopia. PLoS One. 2016;11(4):e0152900.
42. Woimo TT, Yimer WK, Bati T, Gesesew HA. The prevalence and factors associated for anti-tuberculosis treatment non-adherence among pulmonary tuberculosis patients in public health care facilities in South Ethiopia: a cross-sectional study. BMC Public Health. 2017;17(1):269.
43. Tola HH, Holakouie-Naieni K, Tesfaye E, Mansournia MA, Yaseri M. Prevalence of tuberculosis treatment non-adherence in Ethiopia: a systematic review and meta-analysis. Int J Tuberc Lung Dis. 2019;23(6):741-749.
44. Tesfay K, Tesfay S, Nigus E, Gebreyesus A, Gebreegziabiher D, Adane K. More than half of presumptive multidrug-resistant cases referred to a tuberculosis referral laboratory in the Tigray region of Ethiopia are multidrug resistant. Int J Mycobacteriol. 2016;5(3):324-327.
45. Tafess K, Beyen TK, Abera A, Tasew G, Mekit S, Sisay S, et al. Treatment Outcomes of Tuberculosis at Asella Teaching Hospital, Ethiopia: Ten Years' Retrospective Aggregated Data. Front Med (Lausanne). 2018;5:38.
46. Ethiopian Federal Ministry of Health (FMoH). National comprehensive tuberculosis, leprosy and TB/HIV training manual for health care workers. FMoH; Addis Ababa, Ethiopia, 2016.

47. Eshetie S, Gizachew M, Dagnew M, Kumera G, Woldie H, Ambaw F, et al. Multidrug resistant tuberculosis in Ethiopian settings and its association with previous history of anti-tuberculosis treatment: a systematic review and meta-analysis. BMC Infectious Diseases. 2017;17(1):219.
48. Ayalew A, Gashu Z, Anteneh T, Hiruy N, Habte D, Jerene D, et al. Improvement in tuberculosis infection control practice via technical support in two regions of Ethiopia. BMC Infectious Diseases. 2018;18(1):557.
49. World Health Organization. WHO End TB Strategy. WHO; Geneva, Switzerland, 2015.
50. Mhimbira F, Hella J, Maroa T, Kisandu S, Chiryamkubi M, Said K, et al. Home-Based and Facility-Based Directly Observed Therapy of Tuberculosis Treatment under Programmatic Conditions in Urban Tanzania. PLoS One. 2016;11(8):e0161171.
51. Mkopi A, Range N, Lwilla F, Egwaga S, Schulze A, Geubbels E, et al. Adherence to tuberculosis therapy among patients receiving home-based directly observed treatment: evidence from the United Republic of Tanzania. PLoS One. 2012;7(12):e51828.
52. Egwaga S, Mkopi A, Range N, Haag-Arbenz V, Baraka A, Grewal P. Patient-centred tuberculosis treatment delivery under programmatic conditions in Tanzania: a cohort study. BMC Med. 2009;7:80.
53. Oyieng'o D, Park P, Gardner A, Kisang G, Diero L, Sitienei J, et al. Community-based treatment of multidrug-resistant tuberculosis: early experience and results from Western Kenya. Public Health Action. 2012;2(2):38-42.
54. Nackers F, Huerga H, Espié E, Aloo AO, Bastard M, Etard JF, et al. Adherence to self-administered tuberculosis treatment in a high HIV-prevalence setting: a cross-sectional survey in Homa Bay, Kenya. PLoS One. 2012;7(3):e32140.
55. Cremers AL, Gerrets R, Kapata N, Kabika A, Birnie E, Klipstein-Grobusch K, et al. Tuberculosis patients' pre-hospital delay and non-compliance with a longstanding DOT programme: a mixed methods study in urban Zambia. BMC Public Health. 2016;16(1):1130.
56. Serapelwane MG, Davhana-Maselesele M, Masilo GM. Experiences of patients having tuberculosis (TB) regarding the use of Directly Observed Treatment Short-Course (DOTS) in the North West Province, South Africa. Curationis. 2016;39(1):e1-e9.
57. Kaplan R, Caldwell J, Hermans S, Adriaanse S, Mtwisha L, Bekker LG, et al. An integrated community TB-HIV adherence model provides an alternative to DOT for tuberculosis patients in Cape Town. Int J Tuberc Lung Dis. 2016;20(9):1185-91.

58. Gebreweld FH, Kifle MM, Gebremicheal FE, Simel LL, Gezae MM, Ghebreyesus SS, et al. Factors influencing adherence to tuberculosis treatment in Asmara, Eritrea: a qualitative study. J Health Popul Nutr. 2018;37(1):1.
59. Ismail N, Ismail F, Omar SV, Blows L, Gardee Y, Koornhof H, et al. Drug resistant tuberculosis in Africa: Current status, gaps and opportunities. Afr J Lab Med. 2018 Dec 6;7(2):781.
60. World Health Organization. Treatment of tuberculosis: Guidelines for treatment of drug-susceptible tuberculosis and patient care, 2017 update. WHO, Geneva, Switzerland, 2017.
61. Alipanah N, Jarlsberg L, Miller C, Linh NN, Falzon D, Jaramillo E, et al. Adherence interventions and outcomes of tuberculosis treatment: A systematic review and meta-analysis of trials and observational studies. PLoS Med. 2018;15(7):e1002595.
62. DiStefano MJ, Schmidt H. mHealth for Tuberculosis Treatment Adherence: A Framework to Guide Ethical Planning, Implementation, and Evaluation. Glob Health Sci Pract. 2016;4(2):211-21.
63. Elangovan R, Arulchelvan S. A Study on the Role of Mobile Phone Communication in Tuberculosis DOTS Treatment. Indian J Community Med. 2013;38(4):229-33.
64. Lei X, Liu Q, Wang H, Tang X, Li L, Wang Y. Is the short messaging service feasible to improve adherence to tuberculosis care? A cross-sectional study. Trans R Soc Trop Med Hyg. 2013 Oct;107(10):666-8.
65. Nsengiyumva NP, Mappin-Kasirer B, Oxlade O, Bastos M, Trajman A, Falzon D, et al. Evaluating the potential costs and impact of digital health technologies for tuberculosis treatment support. Eur Respir J. 2018;52(5).
66. Subbaraman R, de Mondesert L, Musiimenta A, Pai M, Mayer KH, Thomas BE, et al. Digital adherence technologies for the management of tuberculosis therapy: mapping the landscape and research priorities. BMJ Glob Health. 2018;3(5):e001018.
67. Ngwatu BK, Nsengiyumva NP, Oxlade O, Mappin-Kasirer B, Nguyen NL, Jaramillo E, et al. The impact of digital health technologies on tuberculosis treatment: a systematic review. Eur Respir J. 2018; 51(1): 1701596.
68. Denkinger CM, Grenier J, Stratis AK, Akkihal A, Pant-Pai N, Pai M. Mobile health to improve tuberculosis care and control: a call worth making. Int J Tuberc Lung Dis. 2013;17(6):719-27.
69. Nglazi MD, Bekker LG, Wood R, Hussey GD, Wiysonge CS. Mobile phone text messaging for promoting adherence to anti-tuberculosis treatment: a systematic review. BMC Infect Dis. 2013;13:566.

70. World Health Organization (WHO). Handbook for the use of digital technologies to support tuberculosis medication adherence. Geneva, Switzerland: World Health Organization; 2017.
71. Bediang G, Stoll B, Elia N, Abena JL, Geissbuhler A. SMS reminders to improve adherence and cure of tuberculosis patients in Cameroon (TB-SMS Cameroon): a randomized controlled trial. BMC Public Health. 2018;18(1):583.
72. Fang XH, Guan SY, Tang L, Tao FB, Zou Z, Wang JX, et al. Effect of Short Message Service on Management of Pulmonary Tuberculosis Patients in Anhui Province, China: A Prospective, Randomized, Controlled Study. Med Sci Monit. 2017;23:2465-9.
73. Mohammed S, Glennerster R, Khan AJ. Impact of a daily SMS medication reminder system on tuberculosis treatment outcomes: a randomized controlled trial. PLoS One. 2016;11(11):e0162944.
74. Liu X, Lewis JJ, Zhang H, Lu W, Zhang S, Zheng G et al. Effectiveness of electronic reminders to improve medication adherence in tuberculosis patients: a cluster-randomised trial. PLoS Med. 2015;12(9):e1001876.
75. Iribarren S, Beck S, Pearce PF, Chirico C, Etchevarria M, Cardinale D, et al. TextTB: A Mixed Method Pilot Study Evaluating Acceptance, Feasibility, and Exploring Initial Efficacy of a Text Messaging Intervention to Support TB Treatment Adherence. Tuberc Res Treat. 2013;2013:349394.
76. Onwubiko U, Wall K, Sales RM, Holland DP. Using Directly Observed Therapy (DOT) for latent tuberculosis treatment - A hit or a miss? A propensity score analysis of treatment completion among 274 homeless adults in Fulton County, GA. PLoS One. 2019;14(6):e0218373.
77. Park S, Sentissi I, Gil SJ, Park WS, Oh B, Son AR, et al. Medication Event Monitoring System for Infectious Tuberculosis Treatment in Morocco: A Retrospective Cohort Study. Int J Environ Res Public Health. 2019;16(3).
78. Liu X, Blaschke T, Thomas B, De Geest S, Jiang S, Gao Y, et al. Usability of a Medication Event Reminder Monitor System (MERM) by Providers and Patients to Improve Adherence in the Management of Tuberculosis. Int J Environ Res Public Health. 2017;14(10).
79. Broomhead S, Mars M. Retrospective return on investment analysis of an electronic treatment adherence device piloted in the Northern Cape Province. Telemed J E Health. 2012;18(1):24-31.

80. Thakkar D, Piparva KG, Lakkad SG. A pilot project: 99DOTS information communication technology-based approach for tuberculosis treatment in Rajkot district. Lung India. 2019;36(2):108-111.
81. Lam CK, McGinnis Pilote K, Haque A, Burzynski J, Chuck C, Macaraig M. Using Video Technology to Increase Treatment Completion for Patients With Latent Tuberculosis Infection on 3-Month Isoniazid and Rifapentine: An Implementation Study. J Med Internet Res. 2018;20(11):e287.
82. Garfein RS, Liu L, Cuevas-Mota J, Collins K, Muñoz F, Catanzaro DG, et al. Tuberculosis Treatment Monitoring by Video Directly Observed Therapy in 5 Health Districts, California, USA. Emerg Infect Dis. 2018;24(10):1806-1815.
83. Nguyen TA, Pham MT, Nguyen TL, Nguyen VN, Pham DC, Nguyen BH, et al. Video Directly Observed Therapy to support adherence with treatment for tuberculosis in Vietnam: A prospective cohort study. Int J Infect Dis. 2017;65:85-89.
84. Chuck C, Robinson E, Macaraig M, Alexander M, Burzynski J. Enhancing management of tuberculosis treatment with video directly observed therapy in New York City. Int J Tuberc Lung Dis. 2016;20(5):588-93.
85. Garfein RS, Collins K, Muñoz F, Moser K, Cerecer-Callu P, Raab F, et al. Feasibility of tuberculosis treatment monitoring by video directly observed therapy: a binational pilot study. Int J Tuberc Lung Dis. 2015;19(9):1057-64.
86. Schwartz JK. Pillbox use, satisfaction, and effectiveness among persons with chronic health conditions. Assist Technol 2017;29(4):181-187.
87. e Souza FR, da Silva Santana C. A descriptive study about the use of pillboxes by older adults. Health 2013;05(12):103-109.
88. Sanders MJ, van Oss T. Using daily routines to promote medication adherence in older adults. Am J Occup Ther 2013;67(1):91-99.
89. Hayes TL, Hunt JM, Adami A, Kaye JA. An electronic pillbox for continuous monitoring of medication adherence. Conf Proc IEEE Eng Med Biol Soc 2006;1:6400-6403
90. Zimmerman MA. Empowerment theory: psychological, organizational and community levels of analysis. Handbook of Community Psychology. J. Rappaport and E. Seidman. Boston, MA, Springer US: 43-63, 2000.
91. World Health Organization. Monitoring and evaluating digital health interventions: practical guide to conducting research and assessment. WHO; Geneva, Switzerland, 2016.

92. Wisepill. Somerset West, South Africa. Available at: https://www.wisepill.com/
93. Wisepill Technologies. evriMED500 medication monitoring and reminder system: introduction, Version: 1.333. Wisepill; South Africa, 2019. Available at: https://static1.squarespace.com/static/56224d8fe4b092a567c227aa/t/5c8a542b4e17b635a5f442bf/1552569435055/evriMED500+Product+Handbook+Ver+1.333+14-03-19.pdf
94. Ethiopian Federal Ministry of Health. Health and health related indicators. FMoH; Addis Ababa, Ethiopia, 2016.
95. Morisky DE, DiMatteo MR. Improving the measurement of self-reported medication nonadherence: final response. J Clin Epidemio. 2011;64:258–263
96. Brooke J. The System Usability Scale. Hewlett-Packard, USA, 1986
97. TB CAP. Tool to Estimate Patients Costs. Hague: TB CAP; 2008. http://www.stoptb.org/wg/dots_expansion/tbandpoverty/spotlight.asp
98. EQ 5D. EQ-5D-5L User Guide: basic information on how to use the EQ-5D-5L instrument, Version 2.1. EQ 5D; Rotterdam, The Netherlands, 2015.
99. Quintiles Late Phase. Treatment Satisfaction Questionnaire for Medication (TSQM version 1.4). QLP 2011.
100. World Health Organization. Adherence to long-term therapies: evidence for action. WHO; Geneva, Switzerland, 2003.
101. World Health Organization. WHO guidelines on tuberculosis infection prevention and control, 2019 update. WHO; Geneva, Switzerland, 2019.
102. World Health Organization. Tuberculosis patient cost surveys: a handbook. WHO; Geneva, Switzerland, 2017.
103. World Health Organization. WHO consolidated guidelines on drug-resistant tuberculosis treatment. WHO; Geneva, Switzerland, 2019.
104. World Health Organization. Definitions and reporting framework for tuberculosis – 2013 revision (updated 2014). WHO; Geneva, Switzerland, 2013.
105. Wright A. REDCap: A tool for the electronic capture of research data. J Electron Resour Med Libr. 2016;13:197-201.
