# Supplementary material for: Effect of Digital Medication Event Reminder and Monitor-Observed Therapy vs Standard Directly Observed Therapy on Health-Related Quality of Life and Catastrophic Costs in Patients With Tuberculosis: A Secondary Analysis of a Randomized Clinical Trial
Source: JAMA Netw Open. 2022 Sep 15;5(9):e2230509. doi: 10.1001/jamanetworkopen.2022.30509 (PMC9478770; doi:10.1001/jamanetworkopen.2022.30509)
Supplement: Supplement 2. — eTable. Demographic, behavioral, and clinical factors and risk of low health-related quality of life (n=108) [file jamanetwopen-e2230509-s002.pdf]

Supplemental Online Content

Manyazewal T, Woldeamanuel Y, Fekadu A, Holland DP, Marconi VC. Effect of digital medication event reminder and monitor-observed therapy vs standard directly observed therapy on health-related quality-of-life and catastrophic costs in patients with tuberculosis. *JAMA Netw Open.* 2022;5(9):e2230509. doi:10.1001/jamanetworkopen.2022.30509

eTable. Demographic, behavioral, and clinical factors and risk of low health-related quality of life (n=108)

This supplemental material has been provided by the authors to give readers additional information about their work.

© 2022 Manyazewal T et al. *JAMA Network Open.*

eTable. Demographic, behavioral, and clinical factors and risk of low health-related quality of life (n=108)

Variables	Categories, n	CRR (95%CI)	*p-value*	ARR (95%CI)	*p-value*
**Arm**	Control (56)	1.49 (1.35, 1.65)	***.000****	1.49 (1.35, 1.65)	***.000*****
	Intervention (52)	1		1	
**Sociodemographic characteristics**					
Gender	Female (37)	1.04 (0.92, 1.16)	*.559*		
	Male (71)	1			
Age		1.00 (0.99, 1.01)	***.189****	1.00 (0.99, 1.00)	*.850*
Marital status	Married (50)	0.96 (0.86, 1.07)	*.436*		
	Unmarried (58)	1			
Occupation	No Job (35)	1.02 (0.91, 1.14)	*.783*		
	Have Job (73)	1			
Education	Below Prep. (77)	0.97 (0.86, 1.09)	*.588*		
	Prep. & above (31)	1			
# of cohabitants	≤3 (62)	0.99 (0.88, 1.10)	*.804*		
	≥4 (46)	1			
# bedrooms	1 (67)	1.01 (0.89, 1.13)	*.936*		
	≥2 (41)	1			
Household head	No (47)	0.98 (0.87, 1.09)	*.679*		
	Yes (61)	1			
Residency	Permanent (88)	1.11 (0.95, 1.30)	***.180****	1.06 (0.95, 1.18)	*.292*
	Temporary (20)	1		1	
**Behavioral characteristics**					
Smoking per day	Never (90)	0.91 (0.80, 1.03)	***.122****	1.00 (0.86, 1.17)	*.988*
	Yes (18)	1		1	
Khat	Never (87)	0.91 (0.81, 1.03)	***.143****	1.13 (0.95, 1.34)	*.251*
	Yes (21)	1		1	
Alcohol	Never (67)	0.981 (0.88, 1.09)	*.738*		
	Yes (41)	1			
**Disease conditions**					
HIV	Negative (93)	1.03 (0.87, 1.21)	*.742*		
	Positive (15)	1			
TB treatment	New (98)	1.19 (0.95, 1.49)	***.132****	1.13 (0.94, 1.36)	*.197*
	Relapse (10)	1		1	

CRR, crude relative risk; ARR, adjusted relative risk; CI, confidence interval; n=108 as 1 missing for HIV; Prep: preparatory

© 2022 Manyazewal T et al. *JAMA Network Open.*

© 2022 Manyazewal T et al. *JAMA Network Open.*
