# Supplementary material for: Effect of Digital Medication Event Reminder and Monitor-Observed Therapy vs Standard Directly Observed Therapy on Health-Related Quality of Life and Catastrophic Costs in Patients With Tuberculosis: A Secondary Analysis of a Randomized Clinical Trial
Source: JAMA Netw Open. 2022 Sep 15;5(9):e2230509. doi: 10.1001/jamanetworkopen.2022.30509 (PMC9478770; doi:10.1001/jamanetworkopen.2022.30509)
Supplement: Supplement 3. — Data Sharing Statement [file jamanetwopen-e2230509-s003.pdf]

Manyazewal T, Woldeamanuel Y, Fekadu A, Holland DP, Marconi VC. Effect of digital medication event reminder and monitor-observed therapy vs standard directly observed therapy on health-related quality-of-life and catastrophic costs in patients with tuberculosis: a secondary analysis of a randomized clinical trial. *JAMA Netw Open.* 2022;5(9):e2230509. doi:10.1001/jamanetworkopen.2022.30509

Data Sharing Statement

Data

Data available: Yes
Data types: Other (please specify)
Additional Information: The raw data and informed consent form will be shared by the corresponding author (tsegahunm@gmail.com, tsegahun.manyazewal@aau.edu.et), upon reasonable request, subject to approval by the Institutional Review Board of the College of Health Sciences, Addis Ababa University, Ethiopia.
How to access data: The raw data and informed consent form will be shared by the corresponding author (tsegahunm@gmail.com, tsegahun.manyazewal@aau.edu.et), upon reasonable request, subject to approval by the Institutional Review Board of the College of Health Sciences, Addis Ababa University, Ethiopia.
When available: With publication

Supporting Documents

Document types: None

Additional Information

Who can access the data: Researchers whose proposed use of the data has been approved
Types of analyses: For TB-related research
Mechanisms of data availability: The raw data and informed consent form will be shared by the corresponding author (tsegahunm@gmail.com, tsegahun.manyazewal@aau.edu.et), upon reasonable request, subject to approval by the Institutional Review Board of the College of Health Sciences, Addis Ababa University, Ethiopia.
